# Supplementary material for: Genetic drug target validation using Mendelian randomisation
Source: Nat Commun. 2020 Jun 26;11:3255. doi: 10.1038/s41467-020-16969-0 (PMC7320010; doi:10.1038/s41467-020-16969-0)
Supplement: Supplementary file 1 — Supplementary Information [file 41467_2020_16969_MOESM1_ESM.pdf]

Genetic drug target validation using Mendelian randomization

Schmidt et al.

Contents

1 Supplementary Figures **1**

2 Supplementary Tables **29**

3 Supplementary Methods **44**

4 Supplementary Discussion **45**

5 Supplementary References **48**

1 Supplementary Figures

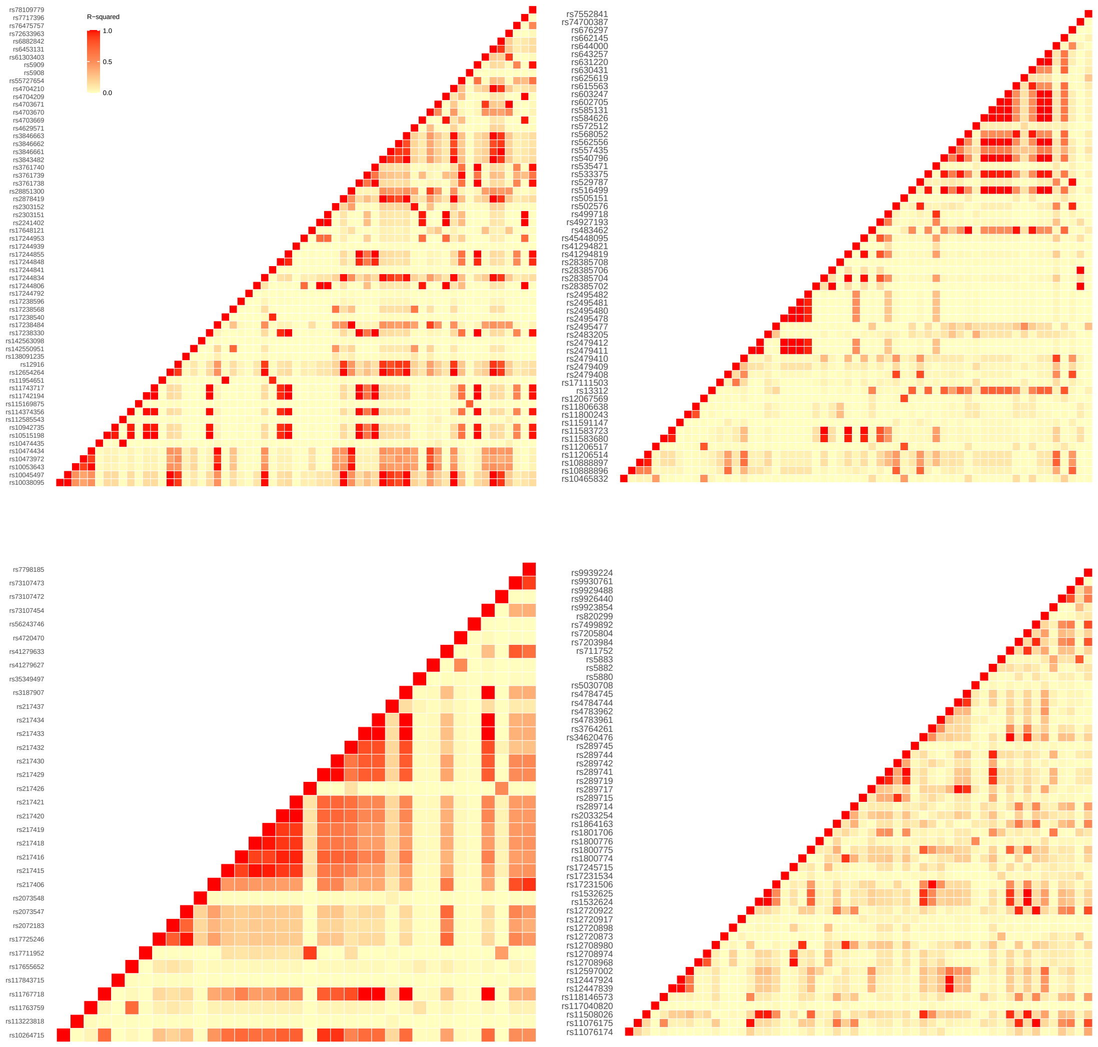

Supplementary Figure 1: Linkage disequilibrium heatmaps of variants with aggregated data on lipids or pQTL associations. Pairwise LD was based on the "EUR" 1000 genomes panel [1]. Left top panel *HMGCR* locus, top right panel *PCSK9* locus, bottom left *NPC1L1* locus, and bottom right *CETP* locus; y-axis labels: rsIDs.

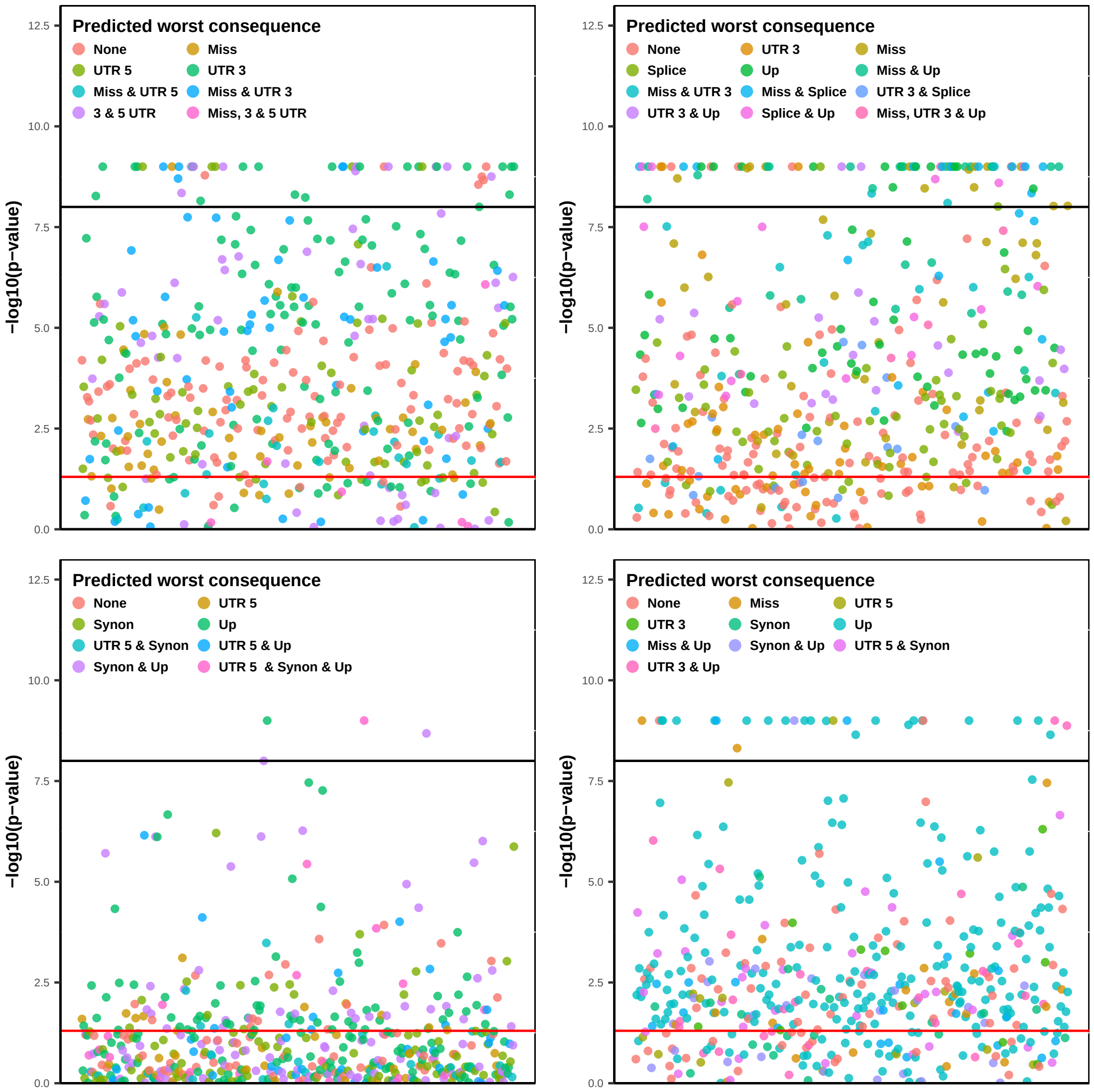

Supplementary Figure 2: A Manhattan plot of 500 repeat-sampled MR analysis using a 4 instruments genetic score. Instruments were taken from the *HMGCR* locus (top left), the *PCSK9* locus (top right panel), *NPC1L1* locus (bottom left), and *CETP* locus (bottom right). Horizontal lines indicate p-value thresholds for 0.05 and 10^{-8}, p-values were truncated at 10^{-9}. Multiplicative random-effects standard error estimates were corrected for LD using the "EUR" 1000 genomes panel [1] and the estimator proposed in [2, 3].

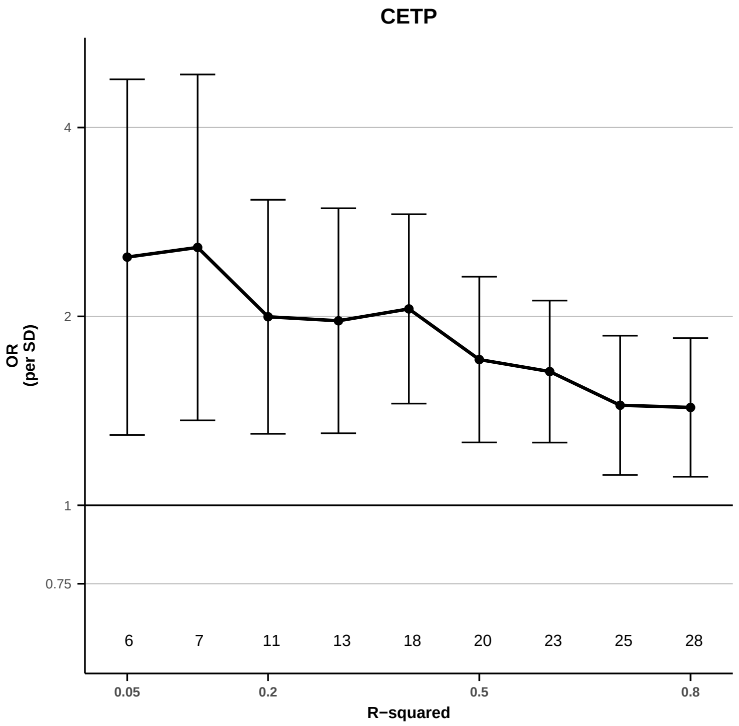

Supplementary Figure 3: Mendelian randomization estimates of the CETP association with CHD weighted by LDL-C under increasingly liberal LD-clumping thresholds. The number of variants at each threshold is provided above the x-axis. Estimates are presented as OR with 95%CI (vertical bars)

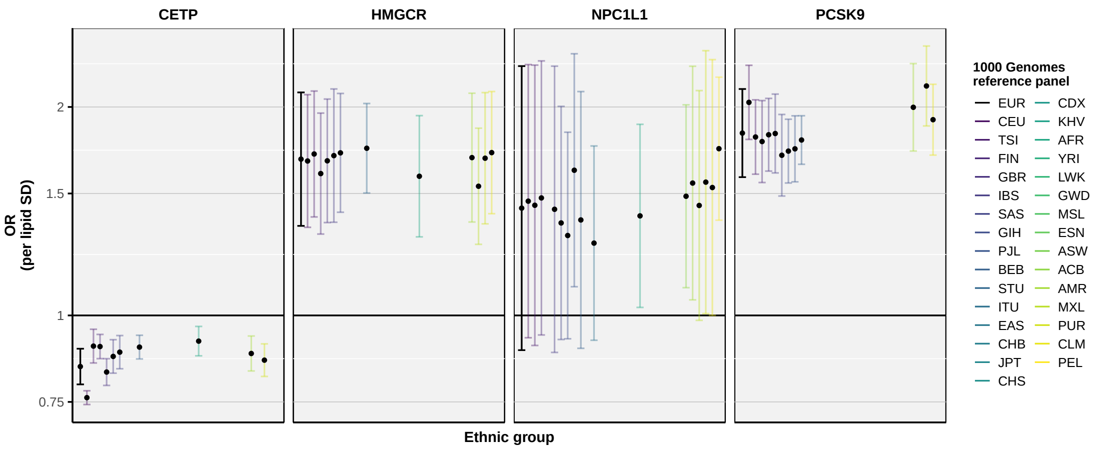

Supplementary Figure 4: Mendelian randomization estimates of lipids weighted associations with CHD, applying LD corrections from different ethnic source populations. To highlight difference between 1000 genomes reference populations fixed effect standard errors were used. Similarly, the candidate variants were kept constant across the different 1000 genomes populations[1] by selecting variants with an R-squared clumping threshold of 0.60 as inferred from the EUR panel. Estimates are given as odd ratios (ORs) and 95% confidence intervals, respectively. Estimates were based on 26 (*CETP*), 10 (*HMGCR*), 11 (*NPC1L1*), and 21 (*PCSK9*) variants, respectively.

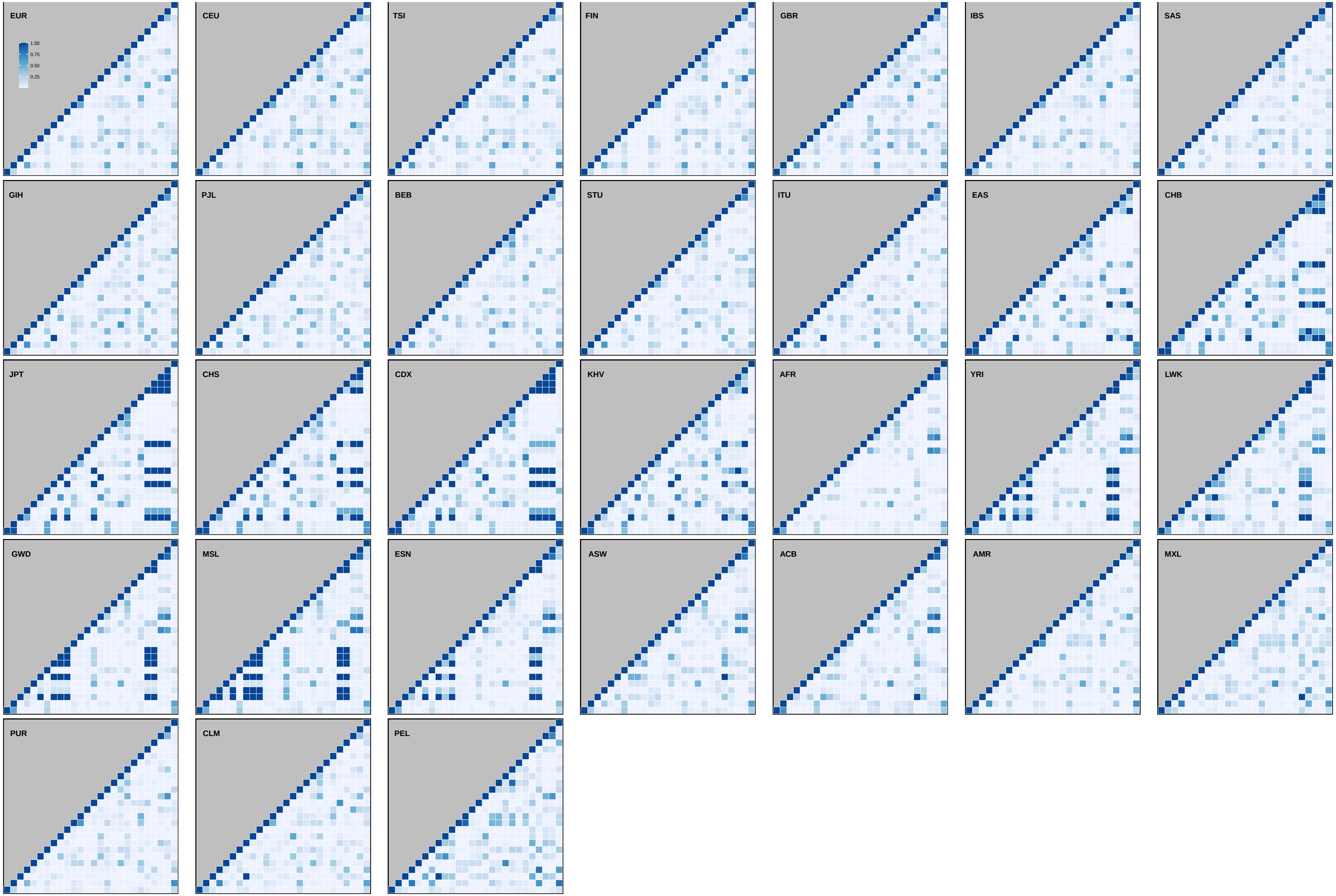

Supplementary Figure 5: Linkage disequilibrium heatmaps from the *CETP* gene using the different 1000 genomes reference panel data[1]. Missing variants are depicted as row and column combinations with background colouring.

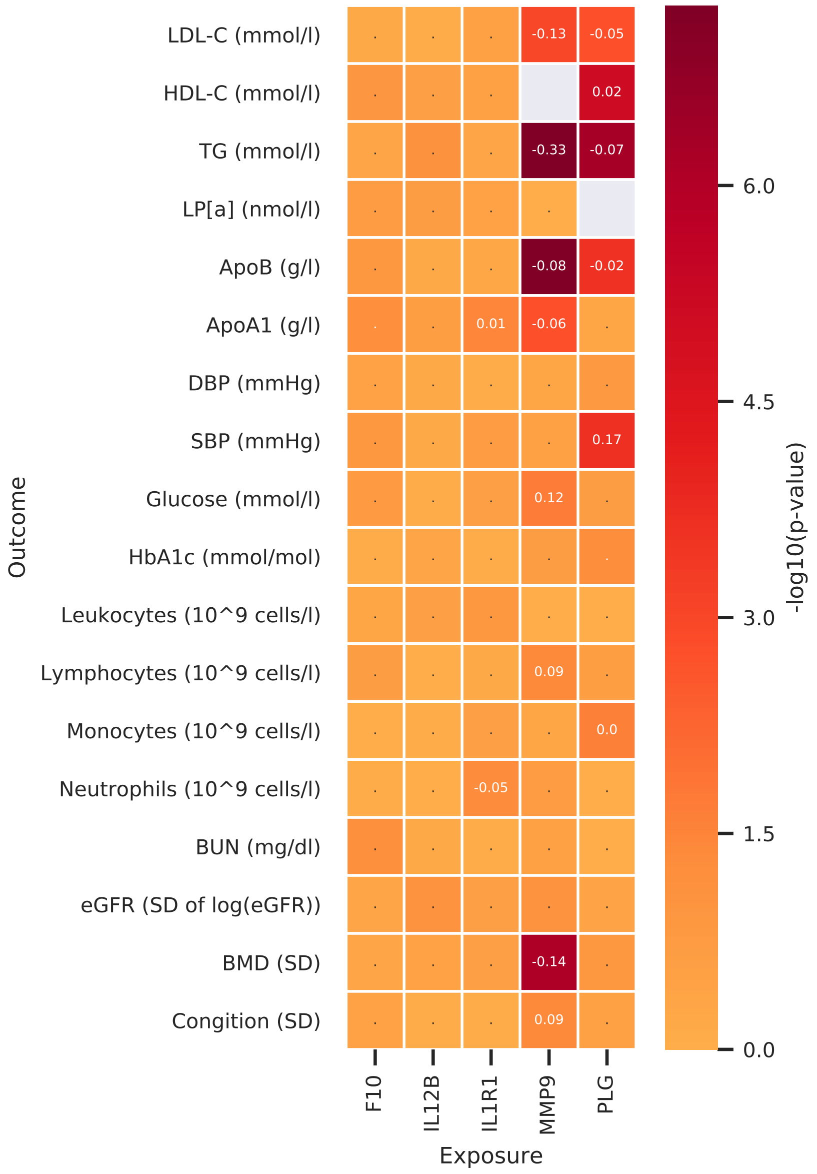

Supplementary Figure 6: Drug target MR effect of increasing protein levels of 5 drug target. Colour intensities are mapped to the $-\log_{10}$ p-value, with grey cell indicating missing data. Point estimates (with direction) is provide for MR results with a p-value smaller than 0.05. pQTL data were extracted from [4], with the outcome data sources described in the methods.

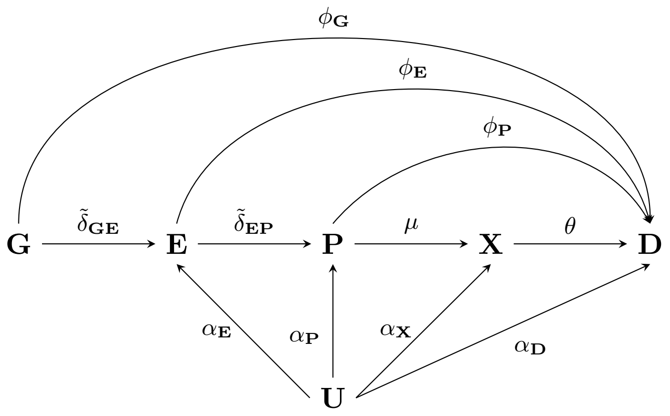

Supplementary Figure 7: A directed acyclic graph depicting a Mendelian randomization study where a genetic variant $\mathbf{G}$ affects mRNA expression $\mathbf{E}$, which in turn affects protein expression $\mathbf{P}$, a downstream biomarker $\mathbf{X}$, and finally disease $\mathbf{D}$. Notice that all non-genetic variables are affect by confounding (a common cause of exposure and disease).

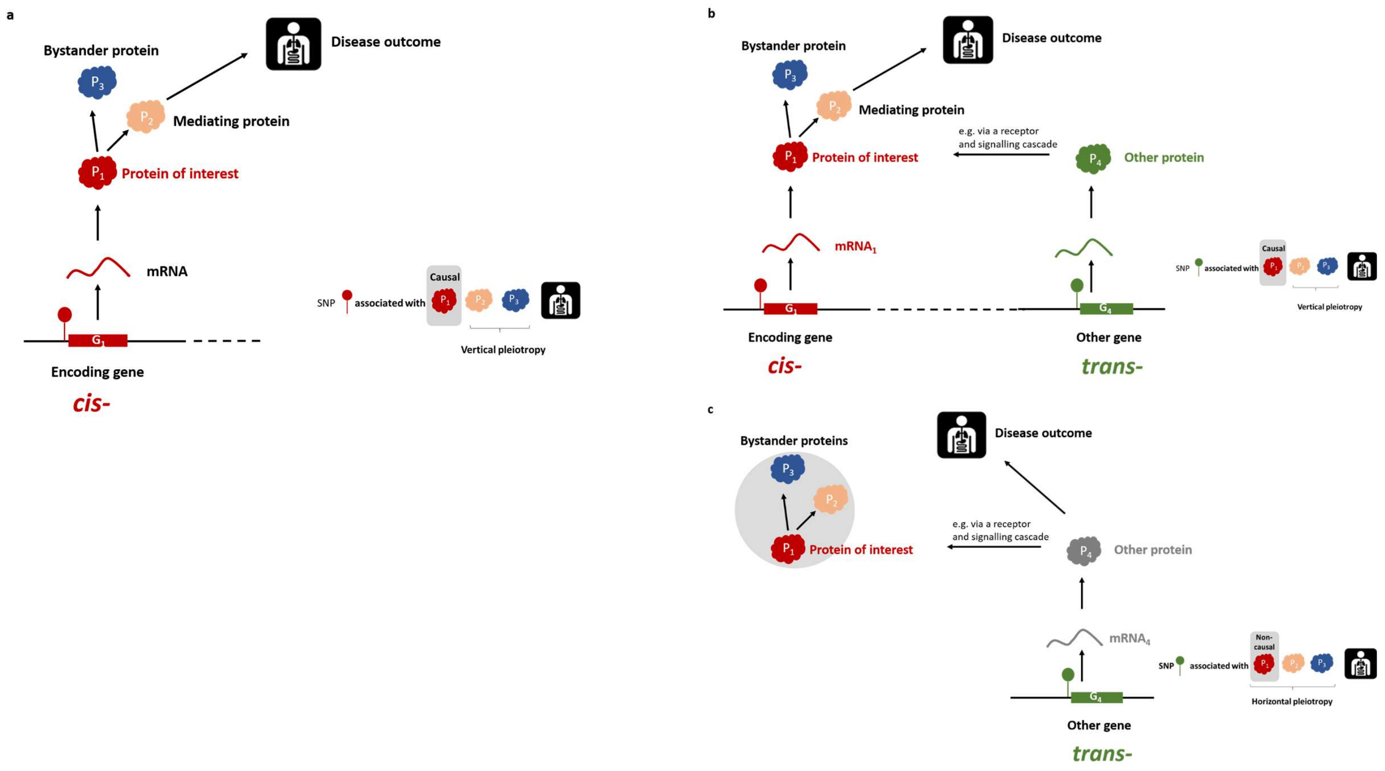

Supplementary Figure 8: Mendelian randomization of a protein exposure using a) *cis*-variants, b) *trans*-variants, affecting a protein cascade $P_4 \rightarrow \ldots \rightarrow P_2$, and c) *trans*-variants erroneously implicating P_1 as the disease causing protein

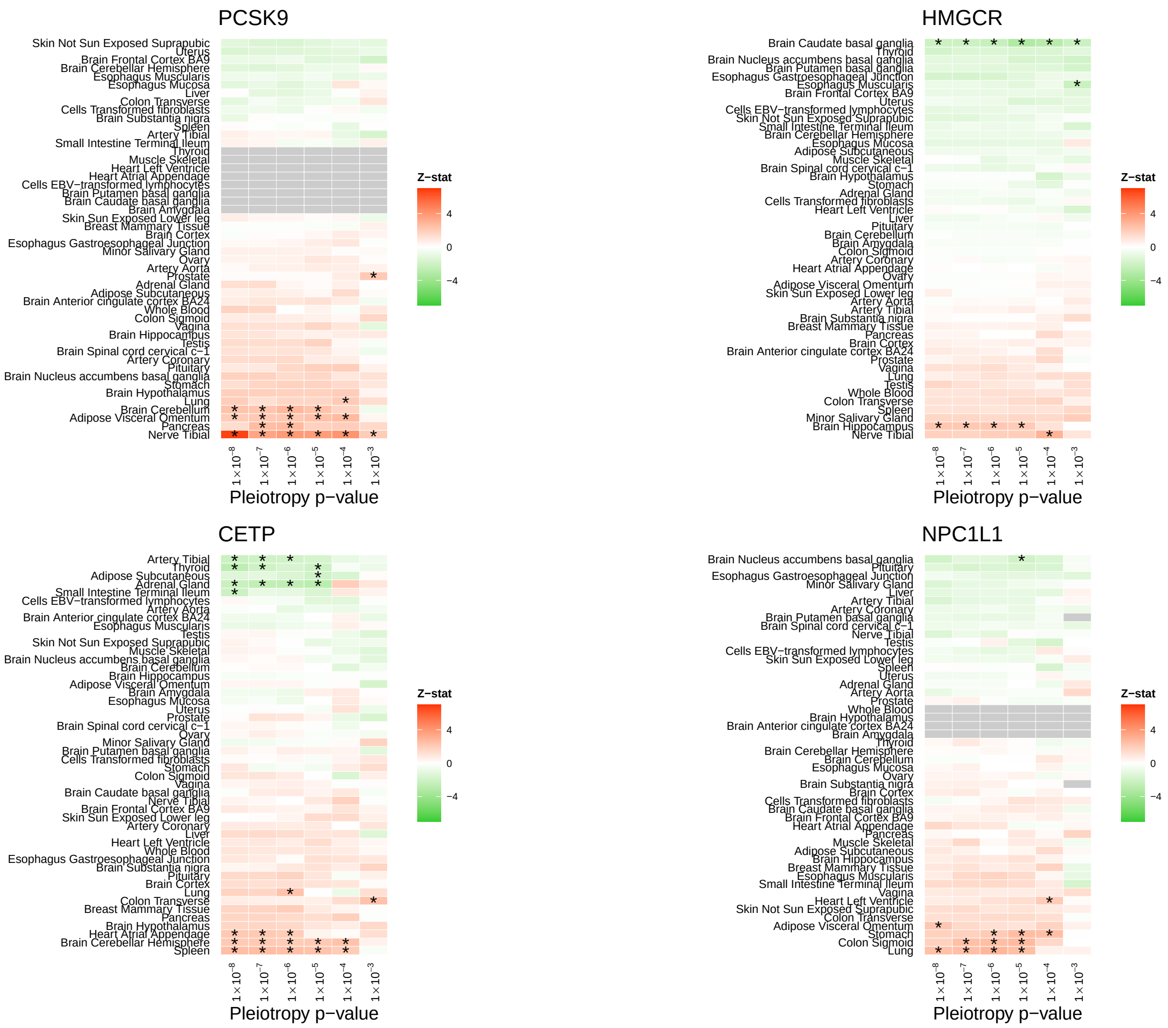

Supplementary Figure 9: Exploring the no-horizontal pleiotropy assumption by pruning variants associated with an non-target eQTL (irrespective of tissue). Z-statistics for the tissue-specific drug target expression on CHD. Estimates were derived selecting variants from within 1 megabase region, clumping to an LD of 0.40 and modeling residual LD using an GLS (IVW) estimator

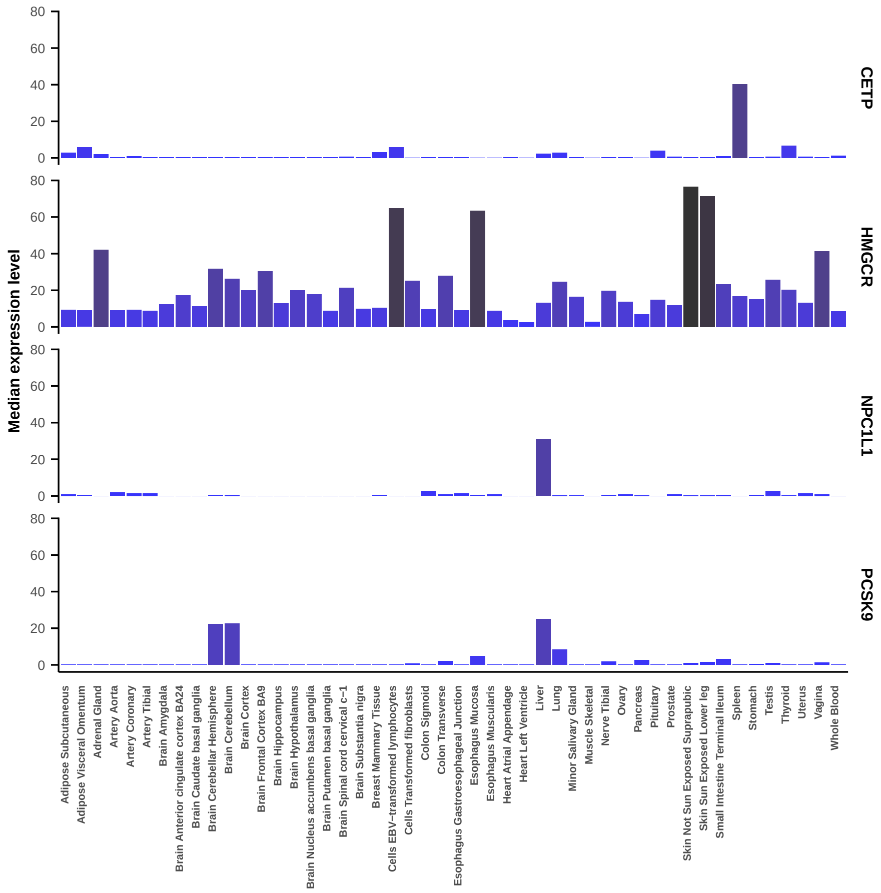

Supplementary Figure 10: Tissue-specific expression level of 4 known drug-targets. N.b. Data were sourced from GTEx [5] and are based on the average expression in 838 donors. Information on variation (e.g., standard deviations) were not provided, nor did we have access to the individual participant data.

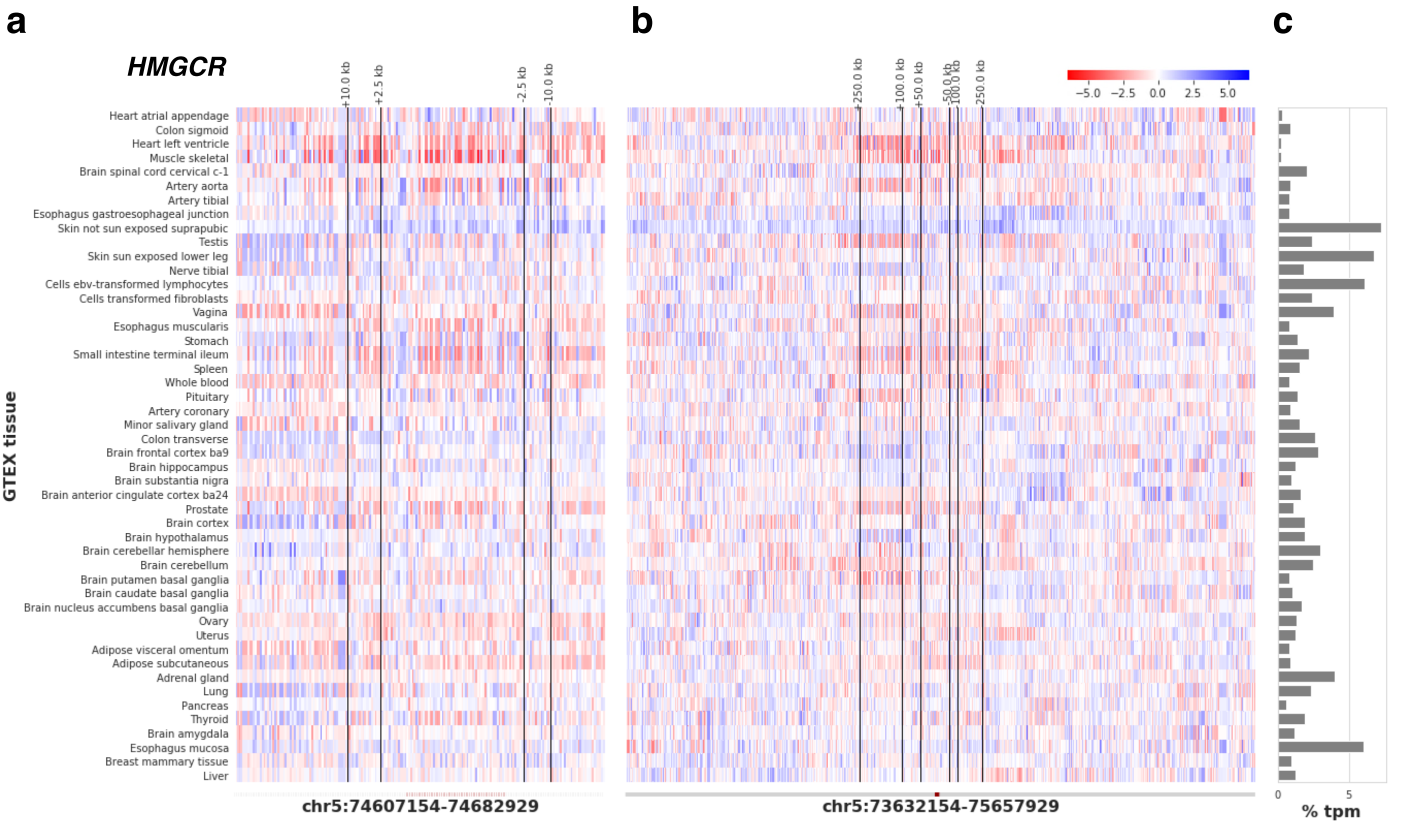

Supplementary Figure 11: eQTL associations in *HMGCR* presented as z-statistics. x-axis indicates the genomic coordinates $\pm$25 kbp (a) and 1Mbp (b), with the gene coloured in maroon. The relative gene expression in transcript per million (%tpm) is shown in c. The alleles are referenced to skin not sun exposed (tissue with the maximum % tpm).

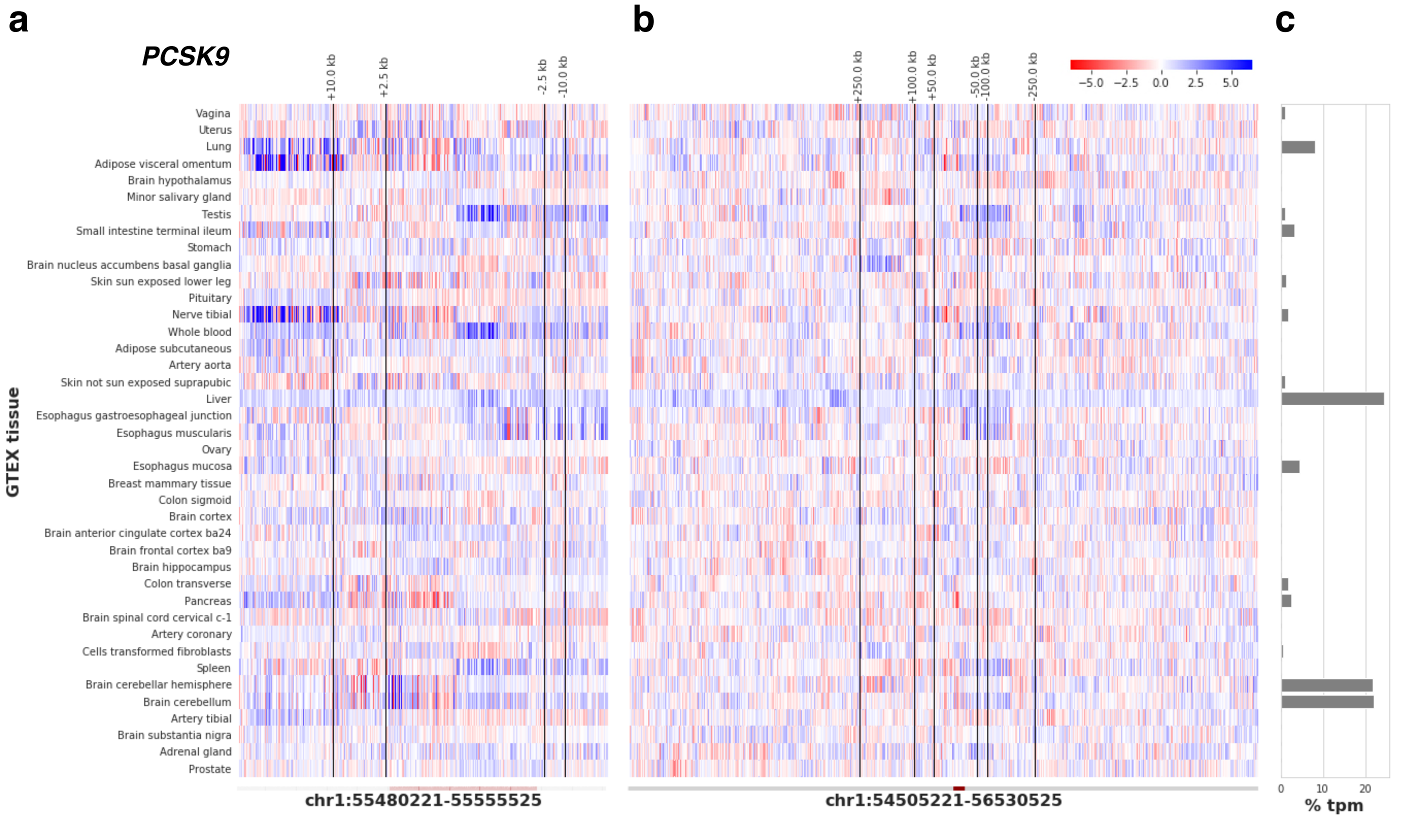

Supplementary Figure 12: eQTL associations in *PCSK9* presented as z-statistics. x-axis indicates the genomic coordinates $\pm$25 kbp (a) and 1Mbp (b), with the gene coloured in maroon. The relative gene expression in transcript per million (%tpm) is shown in c. The alleles are referenced to liver (tissue with the maximum % tpm).

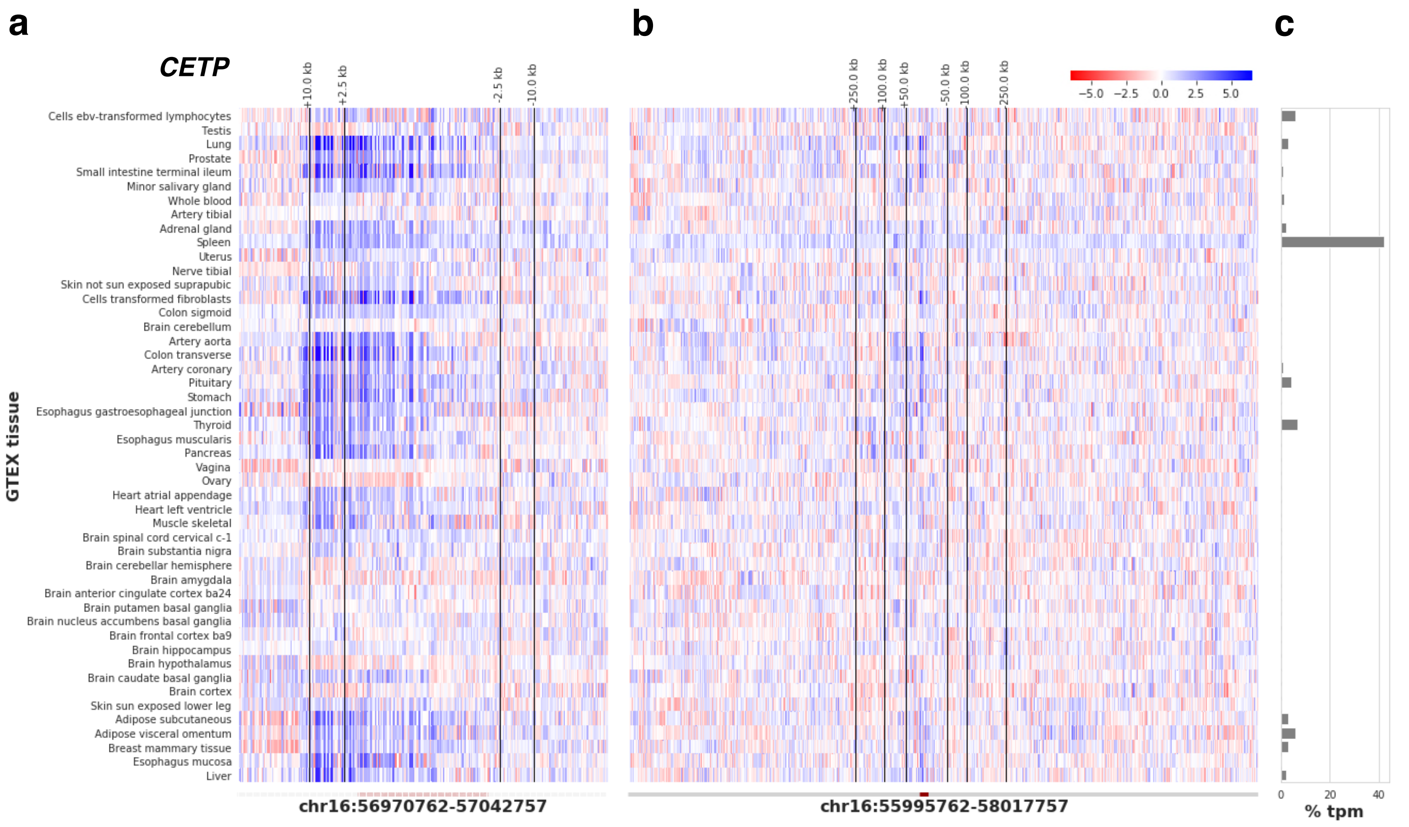

Supplementary Figure 13: eQTL associations in *CETP* presented as z-statistics. x-axis indicates the genomic coordinates ±25 kbp (a) and 1Mbp (b), with the gene coloured in maroon. The relative gene expression in transcript per million (%tpm) is shown in c. The alleles are referenced to spleen (tissue with the maximum % tpm).

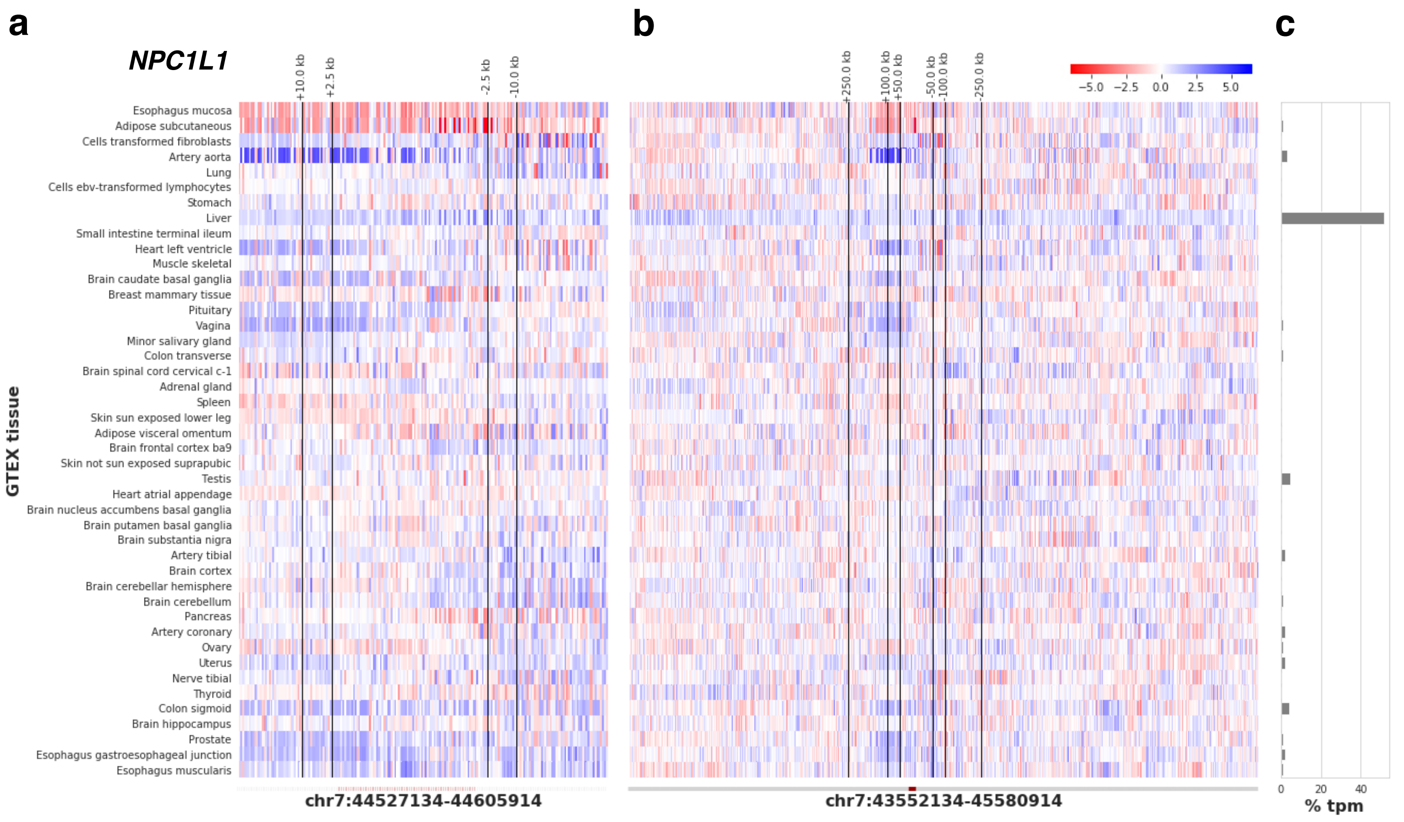

Supplementary Figure 14: eQTL associations in *NPC1L1* presented as z-statistics. x-axis indicates the genomic coordinates ±25 kbp (a) and 1Mbp (b), with the gene coloured in maroon. The relative gene expression in transcript per million (%tpm) is shown in c. The alleles are referenced to liver (tissue with the maximum % tpm).

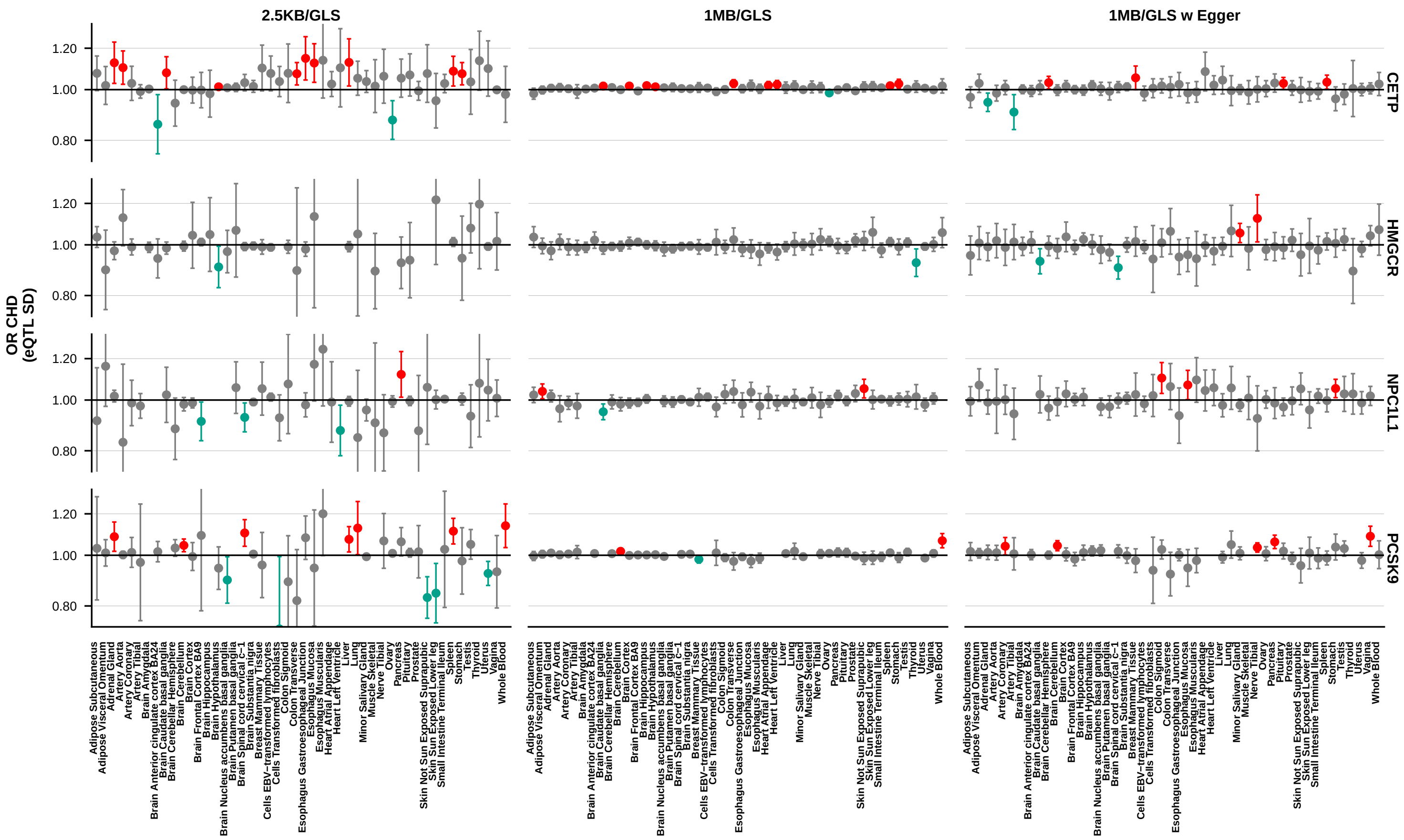

Supplementary Figure 15: Mendelian randomization estimates utilizing GTEx eQTL weights. N.b. Variant were included after clumping on an R-squared threshold of 0.6, with remaining pairwise accounted for using the 1000 genomes "EUR" reference panel and a generalized least squares method with, or without accounting for potential pleiotropy using the Egger correction. Estimates are provided as OR and multiplicative random effects confidence interval (vertical error bars) per SD change in expression level.

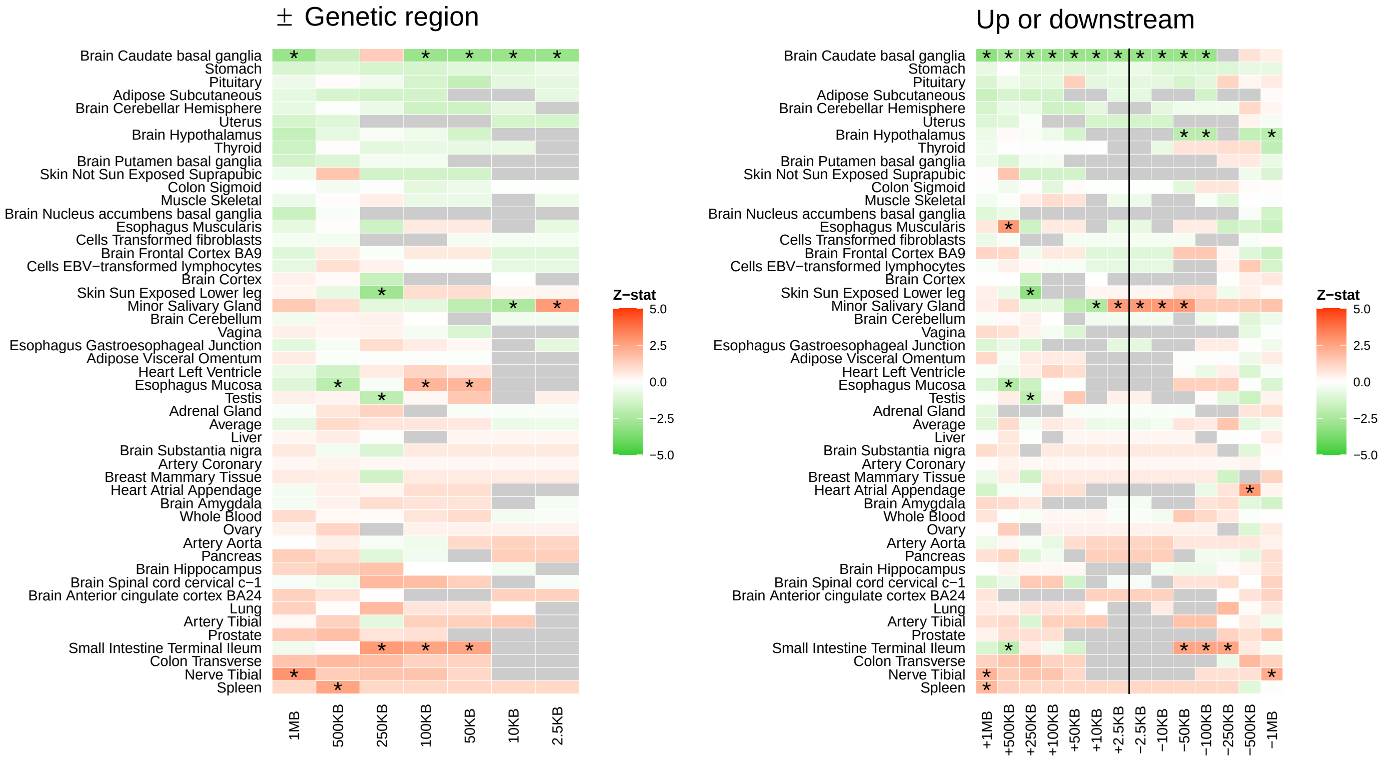

Supplementary Figure 16: Exploring the influence of the genetic region on *HMGCR* expression level associations with CHD, left hand side selecting from ± the genetic region, right hand side selecting from either the upstream region or the downstream region; colours represent z-statistics and stars indicate significant associations at a type 1 error rate of 0.05

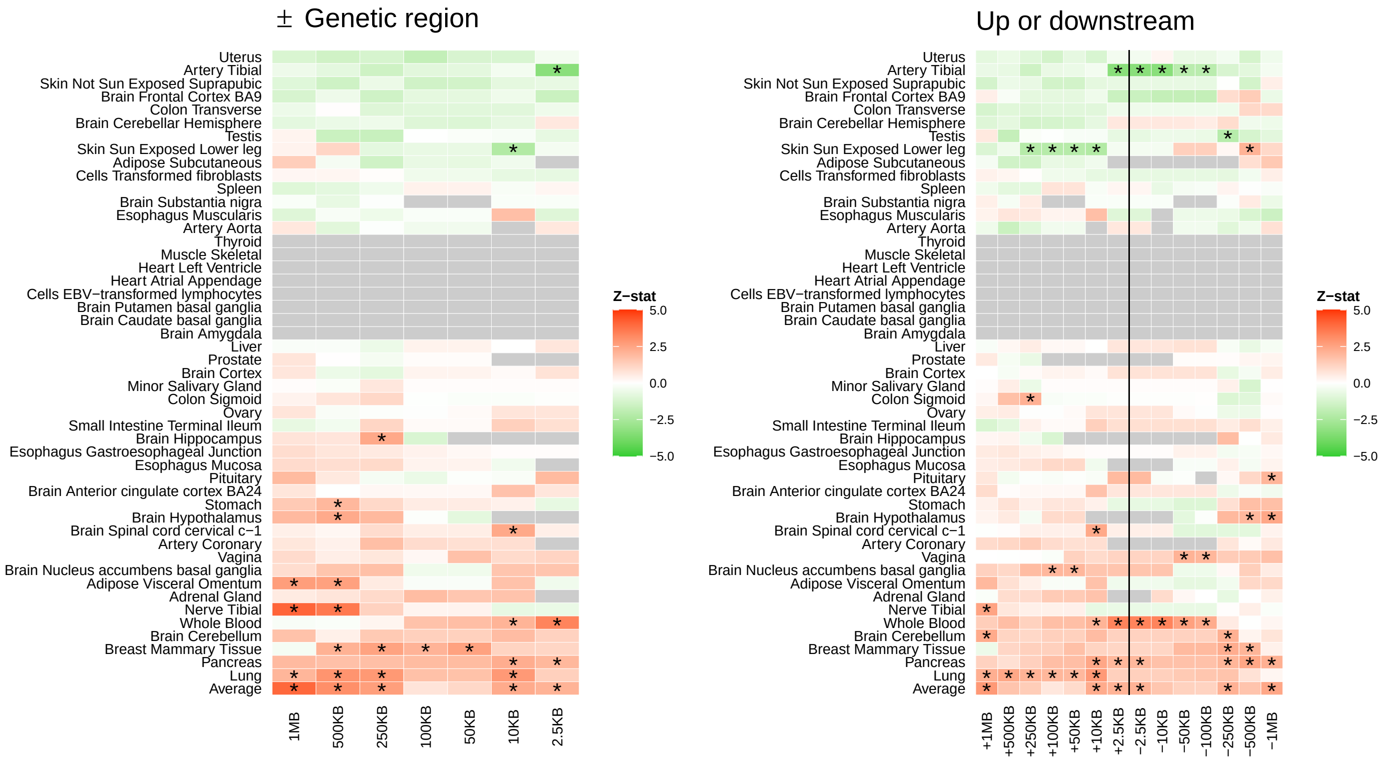

Supplementary Figure 17: Exploring the influence of the genetic region on *PCSK9* expression level associations with CHD, left hand side selecting from ± the genetic region, right hand side selecting from either the upstream region or the downstream region; colours represent z-statistics and stars indicate significant associations at a type 1 error rate of 0.05

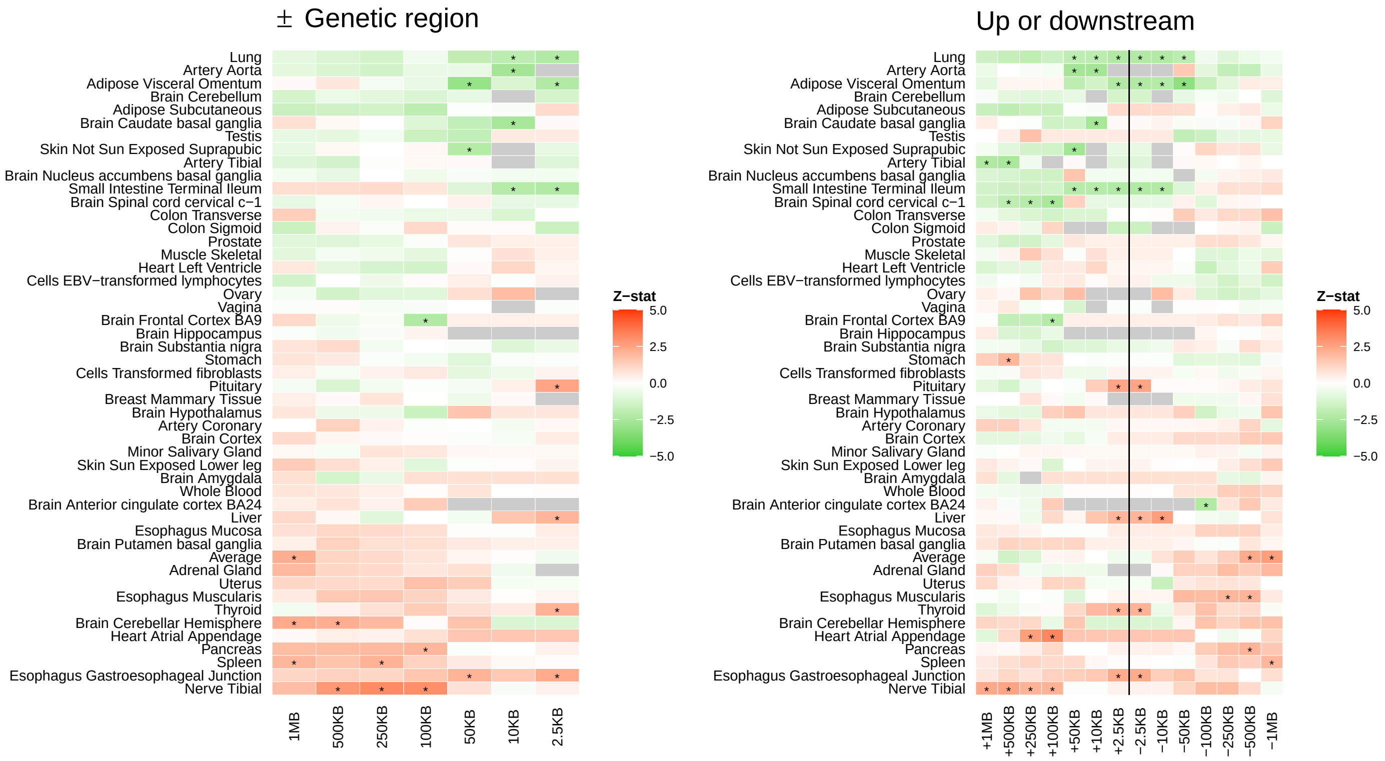

Supplementary Figure 18: Exploring the influence of the genetic region on *CETP* expression level associations with CHD, left hand side selecting from ± the genetic region, right hand side selecting from either the upstream region or the downstream region; colours represent z-statistics and stars indicate significant associations at a type 1 error rate of 0.05

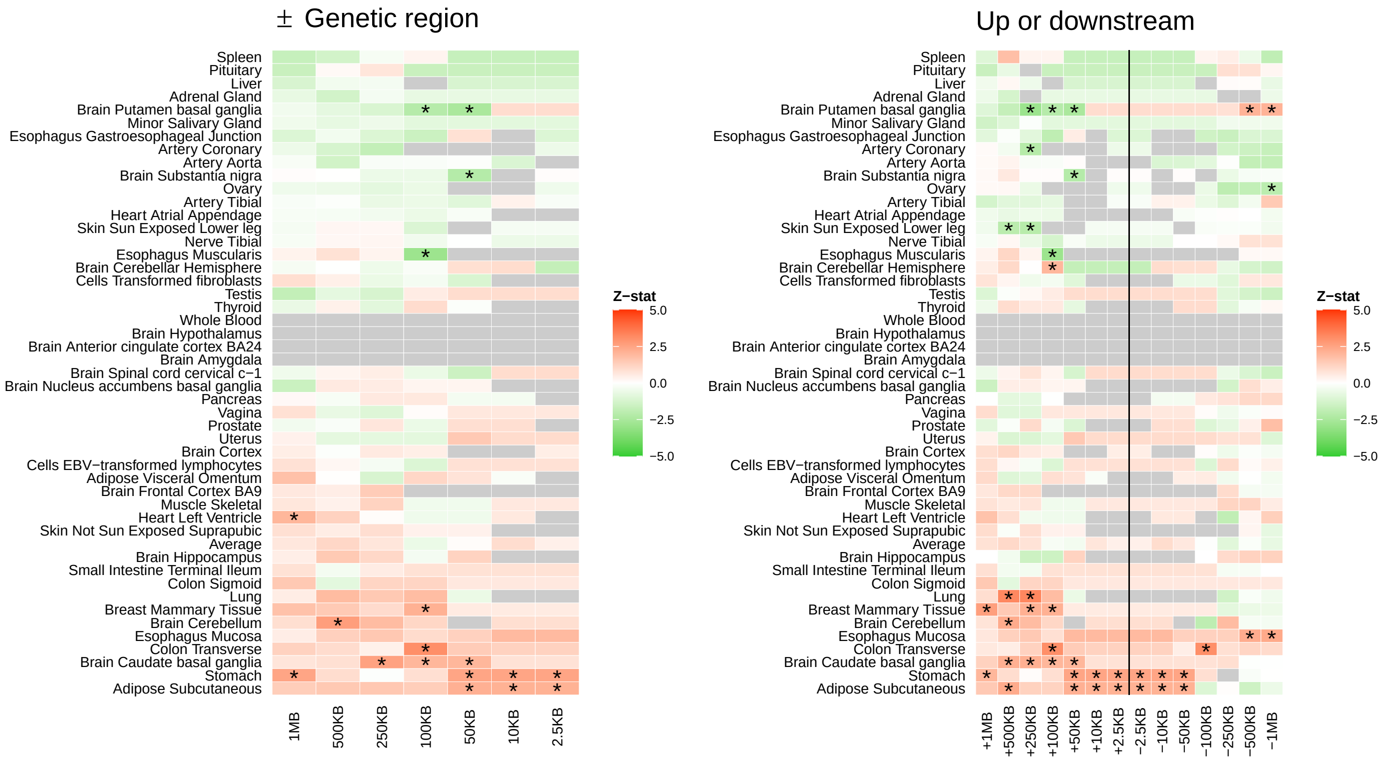

Supplementary Figure 19: Exploring the influence of the genetic region on *NPC1L1* expression level associations with CHD, left hand side selecting from ± the genetic region, right hand side selecting from either the upstream region or the downstream region; colours represent z-statistics and stars indicate significant associations at a type 1 error rate of 0.05

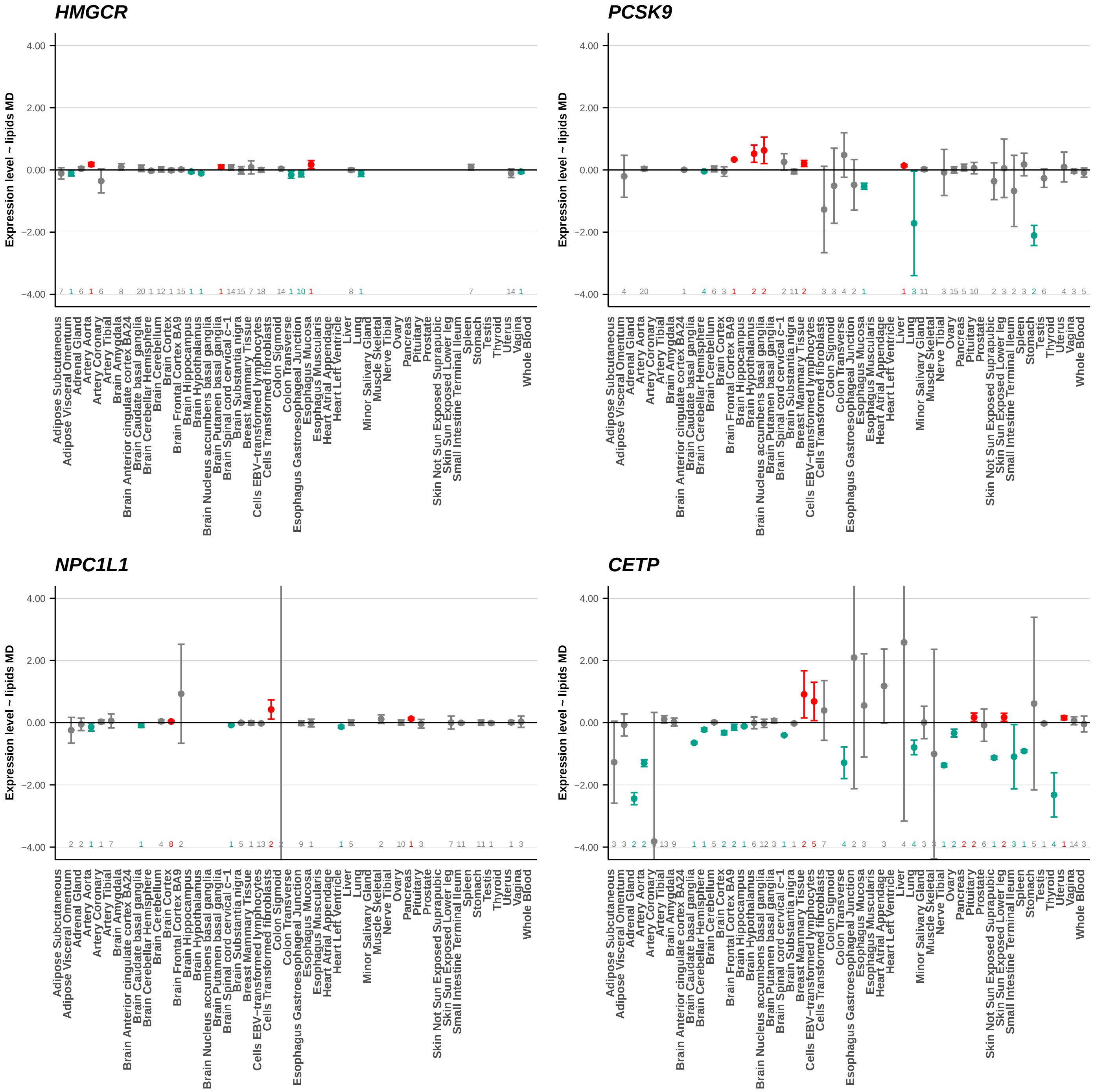

Supplementary Figure 20: Mendelian randomization estimates of the mRNA expression effects on lipid concentration. Estimates are presented as OR with 95%CI (vertial bars). Instruments were taken from the *HMGCR* locus (top left), the *PCSK9* locus (top right panel), *NPC1L1* locus (bottom left), and *CETP* locus (bottom right). eQTL data were available from GTEx [5] and lipids from GLGC [6]; multiplicative random-effects standard error estimates were corrected for LD using the "EUR" 1000 genomes panel [1] and the estimator proposed in [2, 3] with an Egger analytical correction for pleiotropy. The number of variants at each threshold is provided above the x-axis.

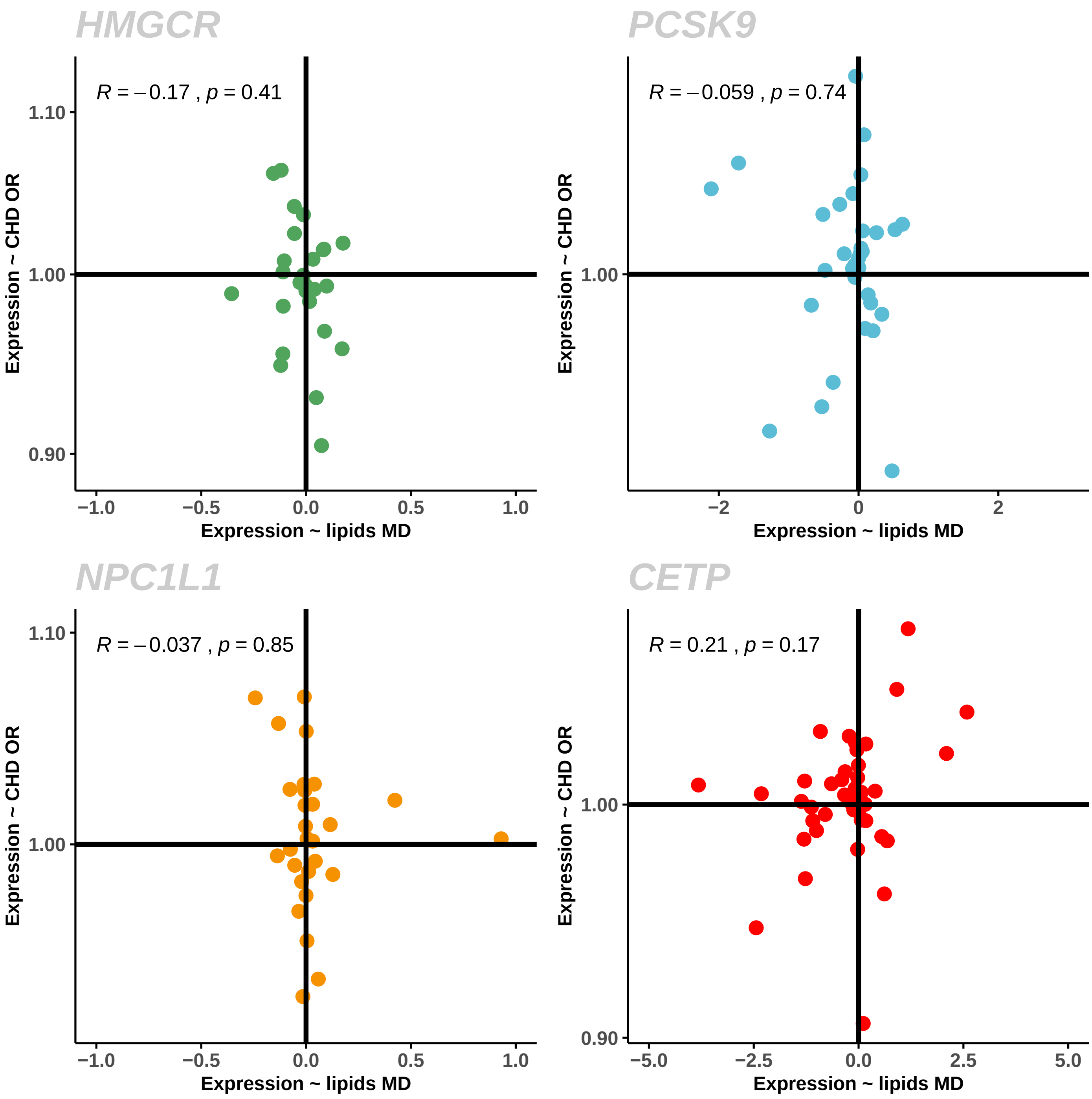

Supplementary Figure 21: Exploring concordance between expression level MR estimates with lipids and with CHD (after Egger correction).

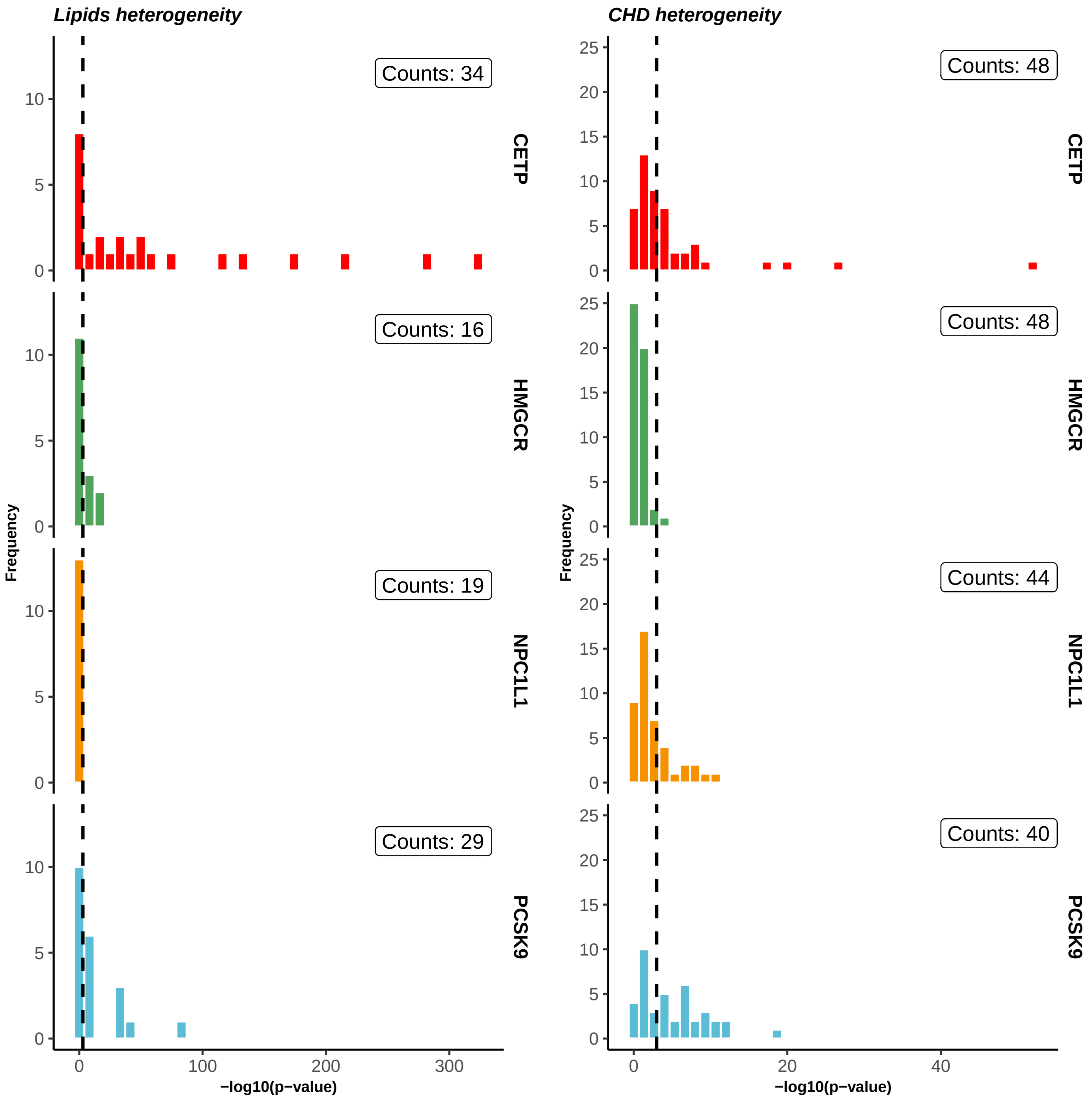

Supplementary Figure 22: Heterogeneity p-values (Q-statistics) of MR-egger adjusted estimates of the tissue specific expression level effects on lipids and CHD. Vertical line indicates a p-value of 0.0010 taking as a conservative indicator of heterogeneity.

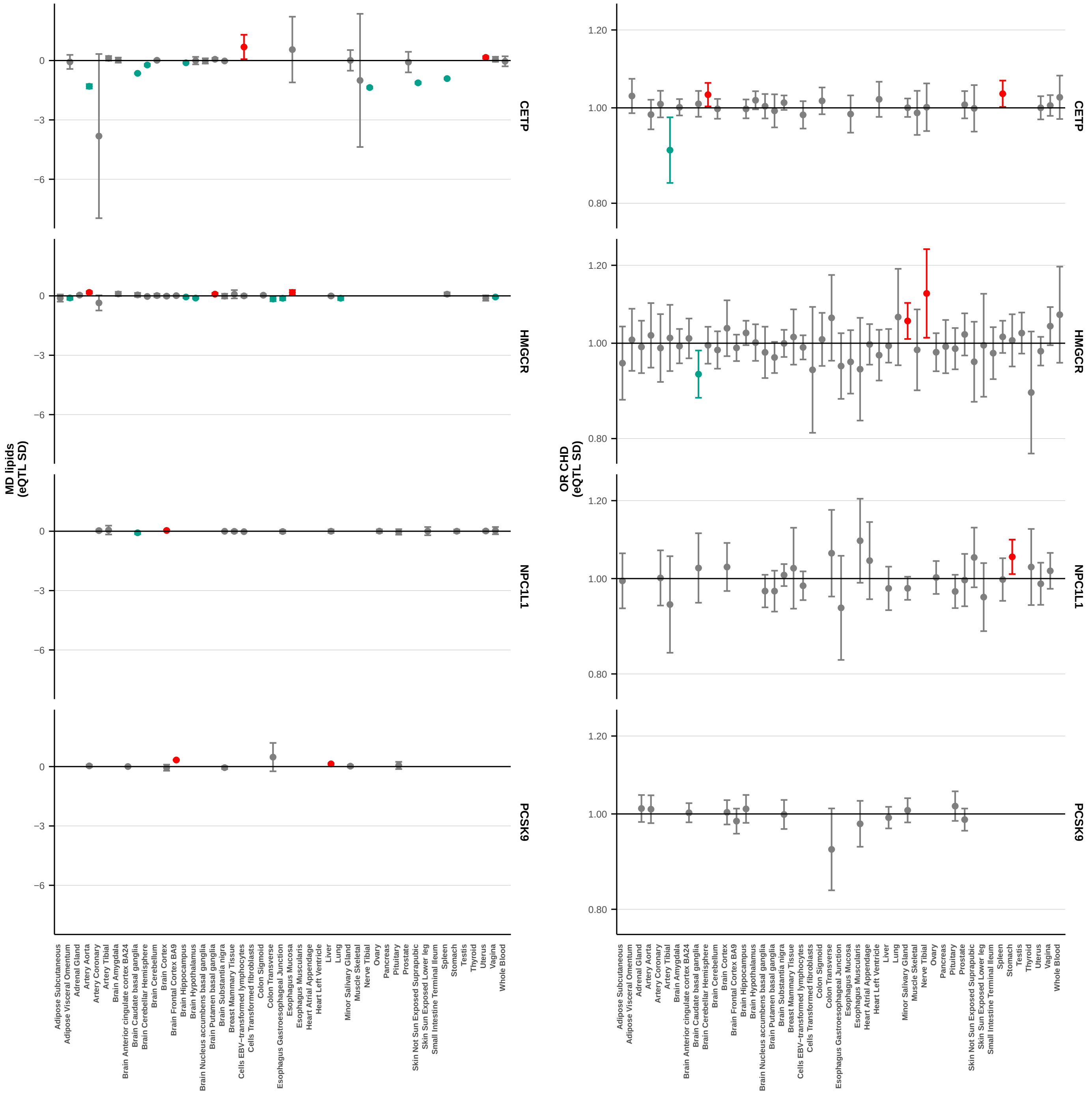

Supplementary Figure 23: The effect of mRNA expression on lipids concentrations and CHD, after excluding heterogeneous tissues. Estimates are provided as OR with 95%CI (vertical bars). The number of variants at each threshold is provided above the x-axis.

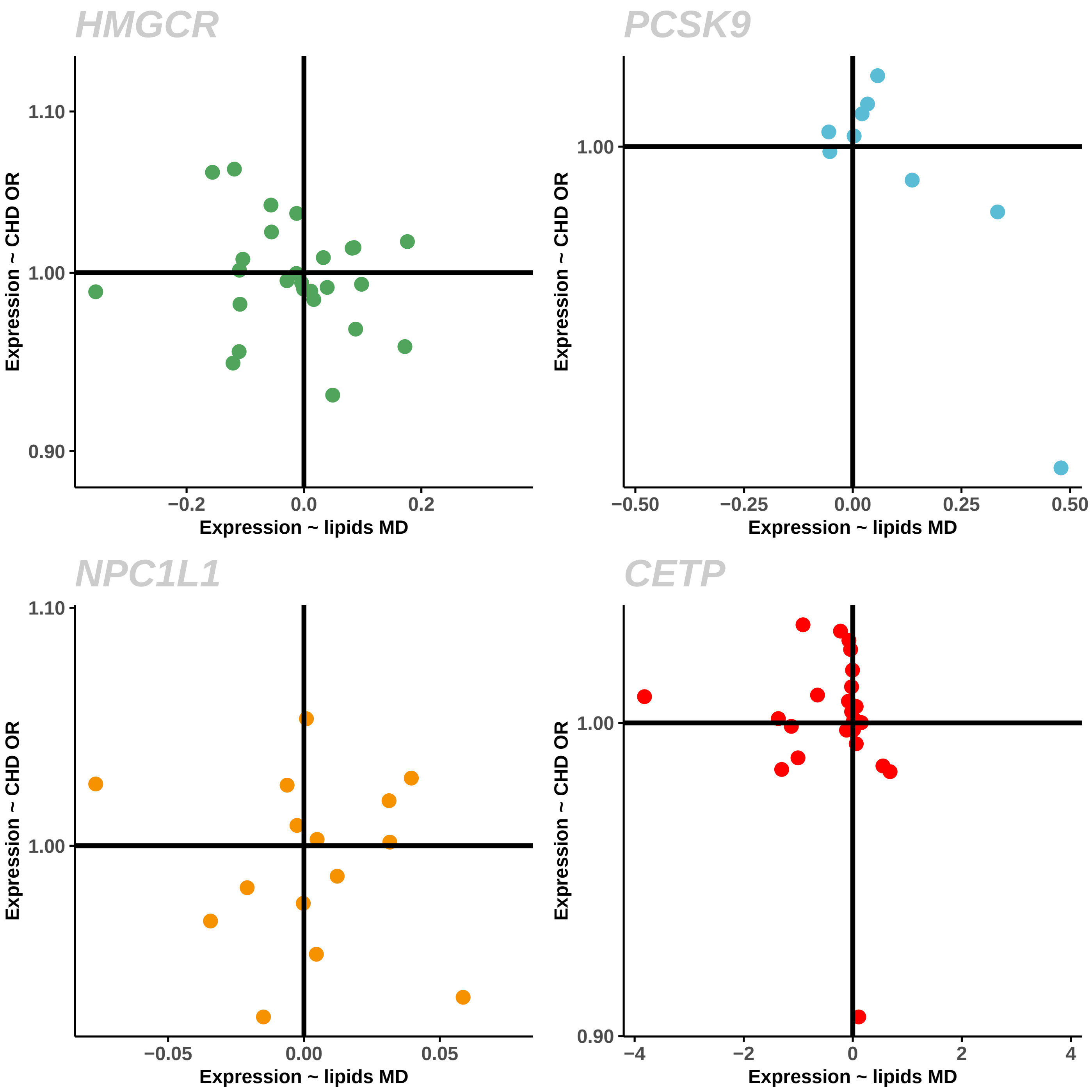

Supplementary Figure 24: Expression effects on lipids and CHD, after excluding heterogeneous tissues (concordance)

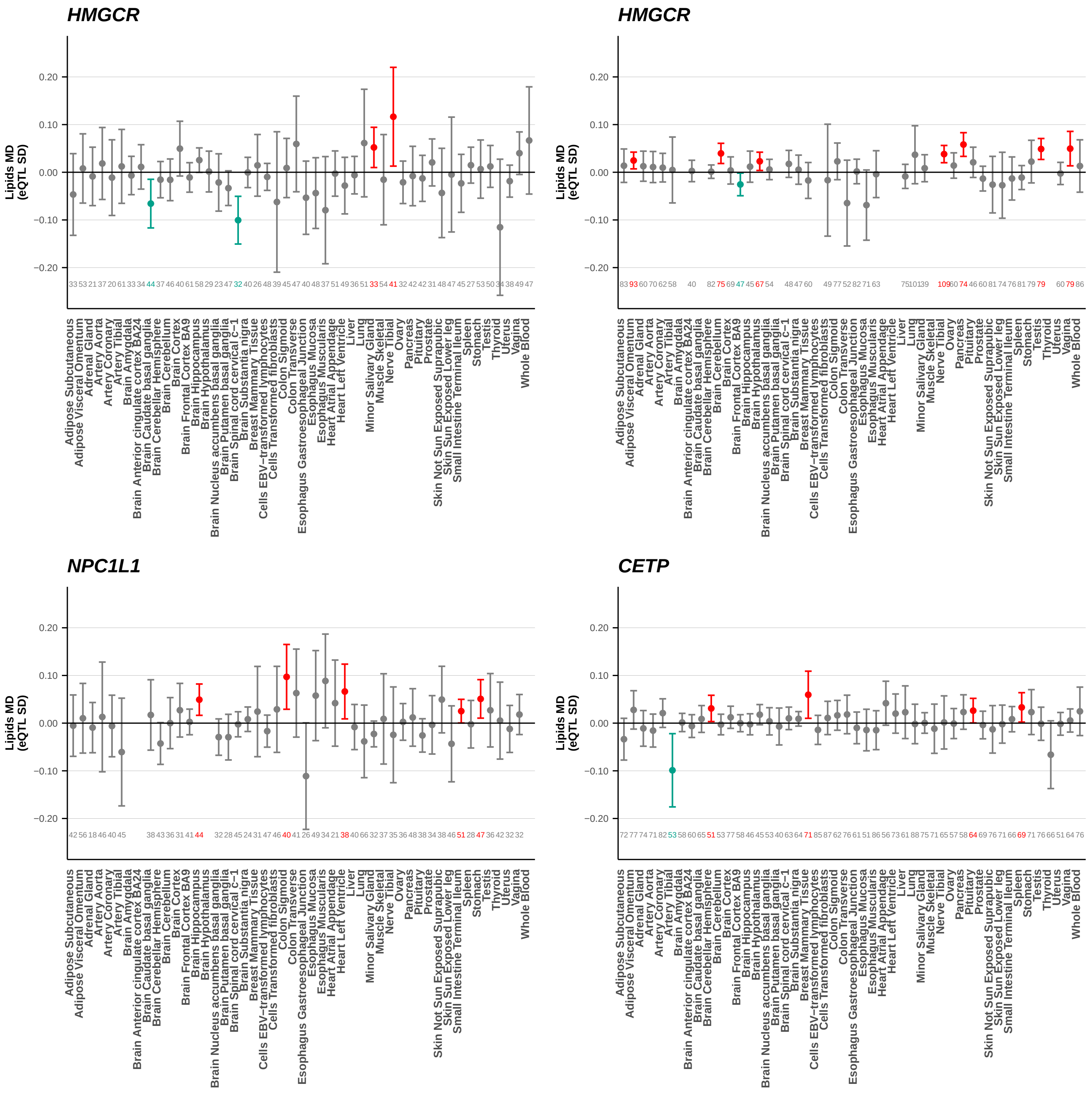

Supplementary Figure 25: Effects of mRNA expression on lipid concentration after removing heterogeneous variants. Estimates are provided as OR with 95%CI (vertical bars). The number of variants at each threshold is provided above the x-axis.

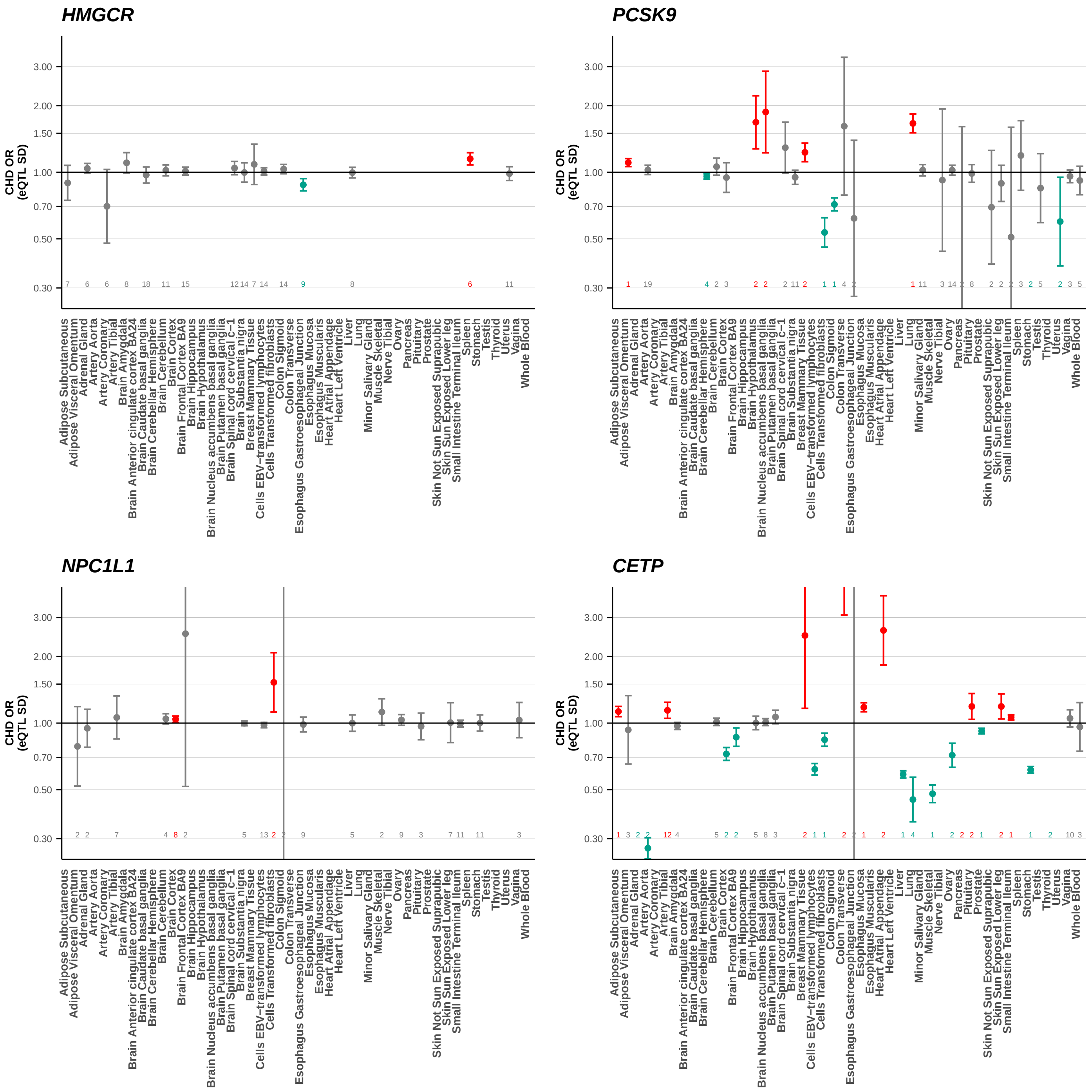

Supplementary Figure 26: mRNA expression effect on CHD effects after removing heterogeneous variants. Estimates are provided as OR with 95%CI (vertical bars). The number of variants at each threshold is provided above the x-axis.

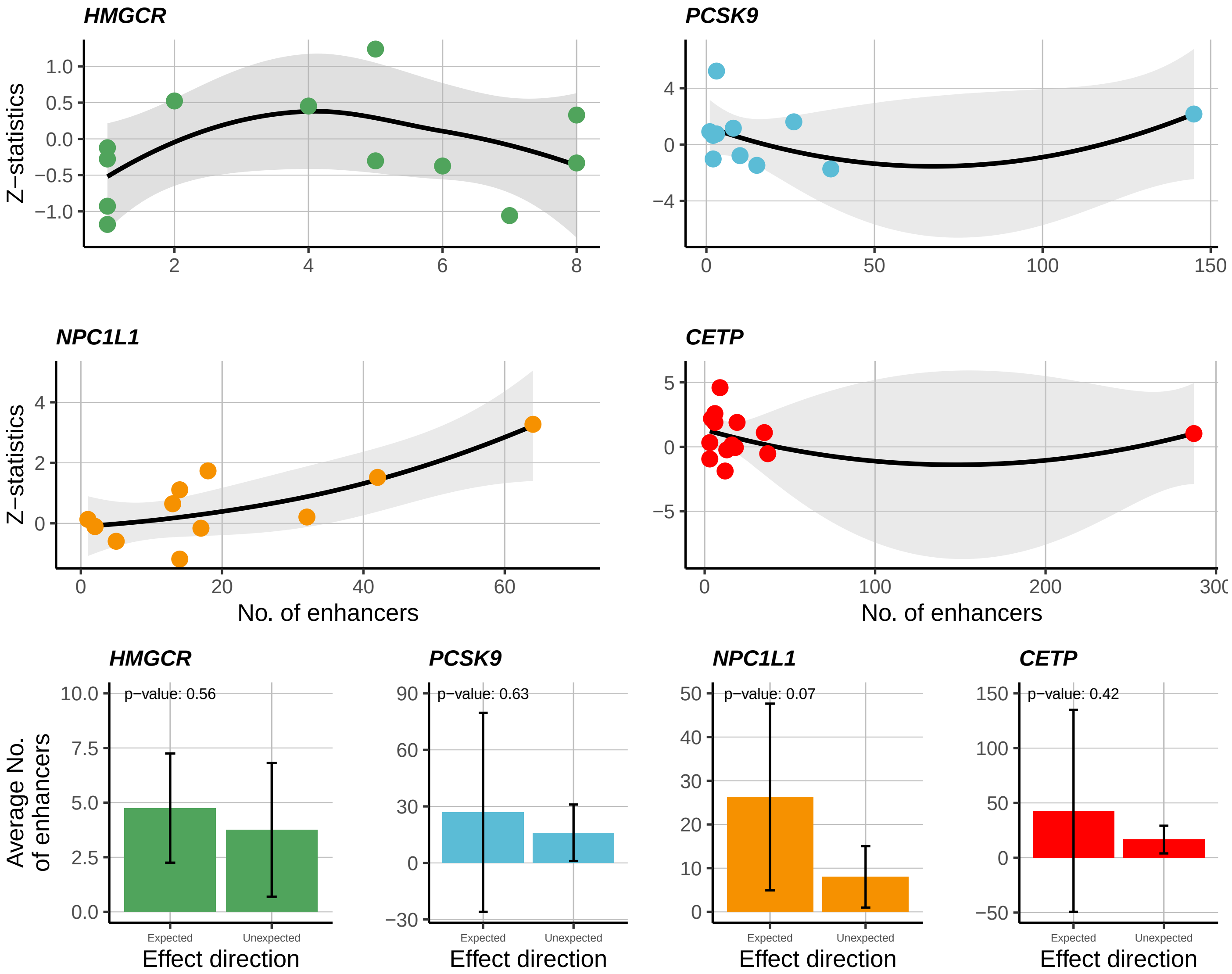

Supplementary Figure 27: The influence of enhancer variants on the expression level association with CHD. P-value were based on two-sample t-tests (degrees of freedom: *HMGCR* (7), *PCSK9* (8), *NPC1L1* (8) *CETP* (9)); vertical error bars indicate the standard deviation (bottom panel), with the grey shaded area indicating the 95%CI (top panel).

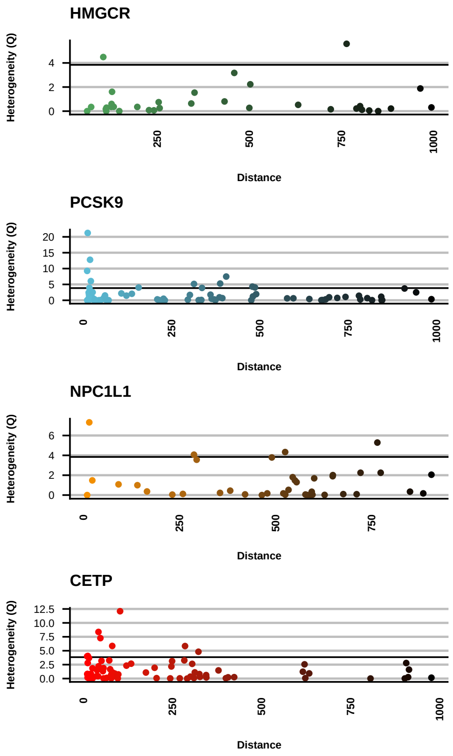

Supplementary Figure 28: Heterogeneity estimates of the tissue specific MR estimates of the expression level effects on CHD ranked by enhancer distance.

2 Supplementary Tables

Supplementary Table 1: Number of variants with lipids or pQTL, and CHD data in a ± 2.5KB region around each locus; stratified by type of mutation (after clumping on $R^2 < 0.80$)

	HMGCR	***PCSK9***	***NPC1L1***	***CETP***
Total number of variants	26	29	16	32
Number of pQTL variants	0	2	0	7
Number of GLGC variants	14	28	16	32
3 prime UTR variant	2	2	0	1
5 prime UTR variant	1	0	1	1
Downstream gene variant	1	0	1	0
Intron variant	17	19	9	20
Missense variant	1	3	0	1
Non coding transcript exon variant	3	1	0	0
Upstream gene variant	1	2	3	8
Splice region variant	0	2	0	0
Synonymous variant	0	0	2	1

Supplementary Table 2: Genetic associations with pQTL, LDL-C, and CHD, in a ± 2.5KB region around the *HMGCR* locus (clumped on $R^2 < 0.80$). GWAS data was extracted from GLGC[6], and CardiogramPlusC4D[7, 8].

**rsID**	**Mean difference per SD LDL**	**SE per SD LDL**	**logOR CHD**	**SE CHD**	**Allele frequency**	**Worst consequence**
rs10473972	NA	NA	-0.023	0.011	NA	intron variant
rs10474435	-0.054	0.015	0.021	0.035	0.011	3 prime UTR variant
rs10515198	-0.060	0.006	-0.039	0.014	0.100	intron variant
rs112585543	NA	NA	-0.007	0.042	NA	intron variant
rs115169875	0.034	0.015	-0.028	0.051	0.021	intron variant
rs12916	-0.073	0.004	-0.039	0.009	0.413	3 prime UTR variant
rs138091235	NA	NA	0.057	0.042	NA	intron variant
rs142550951	NA	NA	-0.007	0.025	NA	intron variant
rs17238540	0.024	0.016	0.026	0.039	0.017	non coding transcript exon variant
rs17238568	-0.032	0.010	-0.023	0.021	0.075	intron variant
rs17238596	NA	NA	-0.001	0.039	NA	intron variant
rs17244792	NA	NA	-0.035	0.056	NA	intron variant
rs17244848	NA	NA	-0.027	0.017	NA	intron variant
rs17244939	0.054	0.020	0.039	0.064	0.013	intron variant
rs17244953	0.005	0.013	-0.018	0.031	0.036	5 prime UTR variant
rs17648121	NA	NA	-0.006	0.033	NA	intron variant
rs2241402	-0.033	0.011	-0.028	0.023	0.051	intron variant
rs2303152	-0.042	0.006	-0.021	0.014	0.110	intron variant
rs3761739	-0.046	0.005	-0.024	0.012	0.152	intron variant
rs3846662	NA	NA	-0.032	0.009	NA	non coding transcript exon variant
rs4629571	NA	NA	-0.010	0.016	NA	upstream gene variant

rs4703670	-0.062	0.004	-0.029	0.010	0.222	downstream gene variant
rs55727654	NA	NA	-0.027	0.014	NA	intron variant
rs5908	0.035	0.014	-0.014	0.055	0.016	missense variant
rs72633963	NA	NA	-0.026	0.014	NA	non coding transcript exon variant
rs76475757	-0.056	0.010	-0.049	0.024	0.061	intron variant

Supplementary Table 3: Genetic associations with pQTL, LDL-C and CHD in a ± 2.5KB region around the *PCSK9* locus (clumped on $R^2 < 0.80$). GWAS data was extracted from Suhre[9], GLGC[6], and Cardiogram-PlusC4D[7, 8].

rsID	Mean difference pQTL	SE pQTL	Mean difference per SD LDL	SE per SD LDL	logOR CHD	SE CHD	Allele frequency	Worst consequence
rs10465832	NA	NA	-0.037	0.010	-0.018	0.019	0.061	intron variant
rs10888896	NA	NA	-0.043	0.005	-0.008	0.013	0.719	intron variant
rs11206514	NA	NA	-0.051	0.004	-0.013	0.011	0.602	intron variant
rs11206517	NA	NA	-0.063	0.014	-0.054	0.027	0.035	intron variant
rs11583680	NA	NA	0.034	0.006	0.037	0.017	0.134	missense variant
rs11591147	-0.642	0.153	0.497	0.018	0.355	0.069	0.021	missense variant
rs11800243	NA	NA	0.014	0.014	0.044	0.030	0.053	intron variant
rs12067569	NA	NA	-0.088	0.010	-0.038	0.023	0.030	intron variant
rs13312	NA	NA	-0.030	0.007	0.004	0.013	0.806	3 prime UTR variant
rs17111503	NA	NA	-0.066	0.004	-0.040	0.011	0.243	upstream gene variant
rs2479409	NA	NA	0.064	0.004	0.037	0.010	0.680	upstream gene variant
rs2483205	NA	NA	0.051	0.005	0.022	0.012	0.450	splice region variant
rs2495477	NA	NA	0.064	0.005	0.036	0.012	0.400	splice region variant
rs2495481	NA	NA	0.008	0.009	0.028	0.022	0.949	intron variant
rs28385708	NA	NA	0.009	0.014	0.024	0.036	0.050	intron variant
rs41294821	NA	NA	0.030	0.020	0.034	0.036	0.026	intron variant
rs4927193	NA	NA	0.035	0.006	0.040	0.016	0.133	intron variant
rs499718	NA	NA	-0.036	0.008	-0.025	0.014	0.824	intron variant
rs505151	0.138	0.114	NA	NA	0.036	0.027	NA	missense variant
rs529787	NA	NA	0.055	0.005	0.001	0.015	0.224	intron variant
rs557435	NA	NA	-0.062	0.007	-0.001	0.014	0.799	intron variant

rs572512	NA	NA	-0.048	0.005	-0.026	0.013	0.354	non coding transcript exon variant
rs585131	NA	NA	-0.064	0.005	-0.015	0.012	0.821	intron variant
rs625619	NA	NA	-0.042	0.005	-0.008	0.012	0.556	intron variant
rs630431	NA	NA	-0.035	0.004	-0.012	0.010	0.707	intron variant
rs644000	NA	NA	0.060	0.006	0.022	0.012	0.362	intron variant
rs662145	NA	NA	-0.005	0.005	0.003	0.011	0.767	3 prime UTR variant
rs74700387	NA	NA	0.033	0.020	0.023	0.031	0.021	intron variant
rs7552841	NA	NA	-0.037	0.004	-0.019	0.011	0.369	intron variant

Supplementary Table 4: Genetic associations with LDL-C and CHD in a ± 2.5KB region around the *NPC1L1* locus (clumped on $R^2 < 0.80$). GWAS data was extracted from GLGC[6], and CardiogramPlusC4D[7, 8].

rsID	Mean difference per SD LDL	SE per SD LDL	logOR CHD	SE CHD	Allele frequency	Worst consequence
rs10264715	-0.022	0.004	-0.007	0.012	0.209	synonymous variant
rs11763759	0.038	0.007	0.024	0.013	0.289	intron variant
rs17655652	0.028	0.004	0.029	0.012	0.294	upstream gene variant
rs2072183	-0.039	0.005	-0.004	0.013	0.234	synonymous variant
rs2073547	-0.048	0.005	-0.004	0.014	0.195	upstream gene variant
rs2073548	-0.014	0.012	0.006	0.023	0.054	upstream gene variant
rs217406	-0.039	0.005	-0.015	0.016	0.173	intron variant
rs217420	-0.022	0.004	-0.007	0.013	0.227	intron variant
rs217426	0.005	0.011	-0.028	0.027	0.029	intron variant
rs217433	-0.020	0.005	-0.008	0.011	0.179	intron variant
rs217437	-0.008	0.004	-0.003	0.012	0.626	downstream gene variant
rs35349497	0.018	0.009	0.008	0.022	0.091	intron variant
rs41279627	0.026	0.013	0.048	0.034	0.047	intron variant
rs41279633	-0.052	0.007	-0.008	0.016	0.143	5 prime UTR variant
rs4720470	0.002	0.010	0.003	0.022	0.067	intron variant
rs73107472	-0.009	0.022	-0.031	0.049	0.014	intron variant

Supplementary Table 5: Genetic associations with pQTL, HDL-C and CHD in a ± 2.5KB region around the *CETP* locus (clumped on $R^2 < 0.80$). GWAS data was extracted from Blauw [10], GLGC[6], and Cardiogram-PlusC4D[7, 8].

rsID	Mean difference pQTL	SE pQTL	Mean difference per SD HDL	SE per SD HDL	logOR CHD	SE CHD	Allele frequency	Worst consequence
rs11076174	-0.236	0.029	0.180	0.008	-0.056	0.020	0.108	intron variant
rs11076175	NA	NA	0.254	0.005	-0.034	0.014	0.206	intron variant
rs117040820	0.356	0.062	-0.181	0.022	0.006	0.061	0.013	intron variant
rs118146573	-0.379	0.023	0.262	0.007	-0.023	0.017	0.122	intron variant
rs12597002	NA	NA	0.085	0.004	-0.017	0.012	0.280	intron variant
rs12708974	NA	NA	-0.008	0.006	0.011	0.016	0.134	intron variant
rs12708980	NA	NA	0.016	0.004	-0.002	0.009	0.351	intron variant
rs12720873	NA	NA	-0.095	0.014	-0.019	0.038	0.026	intron variant
rs12720898	NA	NA	-0.022	0.013	0.017	0.021	0.066	intron variant
rs12720917	0.140	0.024	-0.098	0.006	0.012	0.018	0.140	upstream gene variant
rs1532624	NA	NA	-0.204	0.004	0.027	0.011	0.426	intron variant
rs17231506	NA	NA	-0.238	0.004	0.027	0.011	0.291	upstream gene variant
rs17231534	NA	NA	0.016	0.012	0.015	0.024	0.045	5 prime UTR variant
rs17245715	NA	NA	-0.001	0.008	0.001	0.017	0.124	upstream gene variant
rs1800775	0.267	0.014	-0.202	0.004	0.031	0.010	0.488	upstream gene variant
rs1800776	NA	NA	0.057	0.010	-0.007	0.024	0.065	upstream gene variant
rs1801706	NA	NA	-0.064	0.008	-0.002	0.014	0.193	3 prime UTR variant
rs1864163	-0.321	0.017	0.225	0.004	-0.030	0.012	0.270	intron variant
rs289714	NA	NA	-0.214	0.005	0.014	0.014	0.798	intron variant
rs289715	NA	NA	0.134	0.006	-0.060	0.050	0.878	intron variant
rs289717	NA	NA	0.085	0.005	-0.013	0.012	0.326	intron variant

rs289719	NA	NA	0.113	0.004	-0.023	0.011	0.670	intron variant
rs289745	NA	NA	-0.028	0.004	-0.004	0.013	0.604	upstream gene variant
rs4783961	0.133	0.015	-0.100	0.004	0.022	0.011	0.469	upstream gene variant
rs4783962	NA	NA	-0.075	0.004	0.010	0.011	0.770	upstream gene variant
rs5030708	NA	NA	0.079	0.016	-0.014	0.037	0.034	intron variant
rs5880	NA	NA	0.307	0.009	-0.025	0.024	0.052	missense variant
rs5883	NA	NA	-0.115	0.008	-0.024	0.026	0.056	synonymous variant
rs820299	NA	NA	-0.064	0.004	0.005	0.011	0.637	intron variant
rs9923854	NA	NA	-0.084	0.010	-0.022	0.019	0.127	intron variant
rs9929488	NA	NA	0.176	0.004	-0.024	0.012	0.289	intron variant
rs9930761	NA	NA	-0.062	0.008	-0.014	0.023	0.073	intron variant

Supplementary Table 6: The *HMGCR* lipids - CHD effect, comparing the GLS estimates to functional specific MR estimates

Type of variant selection	***nSNPs***	***Fixed effect***	***Random effects***
GLS	10	1.68 (1.35;2.10)	1.68 (1.35;2.10)
3 prime UTR variant	2	1.68 (1.32;2.12)	1.68 (1.20;2.34)
intron variant	5	1.80 (1.30;2.48)	1.80 (1.30;2.48)
non coding transcript exon variant	1	2.99 (0.13;71.42)	NA
downstream gene variant	1	1.61 (1.17;2.22)	NA
missense variant	1	0.67 (0.03;14.02)	NA
upstream gene variant	NA	NA	NA
splice region variant	NA	NA	NA
5 prime UTR variant	NA	NA	NA
synonymous variant	NA	NA	NA
Miss & splice	1	0.67 (0.03;14.02)	NA
3- & 5-UTR & intron & nc exon	8	1.66 (1.32;2.08)	1.66 (1.32;2.08)
Down & upstream	1	1.61 (1.17;2.22)	NA

Supplementary Table 7: The *PCSK9* lipids - CHD effect, comparing the GLS estimates to functional specific MR estimates

Type of variant selection	*nSNPs*	*Fixed effect*	*Random effects*
GLS	21	1.83 (1.58;2.12)	1.83 (1.55;2.17)
3 prime UTR variant	NA	NA	NA
intron variant	16	1.53 (1.25;1.88)	1.53 (1.24;1.90)
non coding transcript exon variant	1	1.71 (1.02;2.88)	NA
downstream gene variant	NA	NA	NA
missense variant	1	2.04 (1.56;2.67)	NA
upstream gene variant	1	1.78 (1.31;2.41)	NA
splice region variant	2	1.70 (1.21;2.40)	1.70 (1.21;2.40)
5 prime UTR variant	NA	NA	NA
synonymous variant	NA	NA	NA
Miss & splice	3	1.91 (1.54;2.36)	1.91 (1.54;2.36)
3- & 5-UTR & intron & nc exon	17	1.64 (1.35;2.00)	1.64 (1.31;2.06)
Down & upstream	1	1.78 (1.31;2.41)	NA

Supplementary Table 8: The *NPC1L1* lipids - CHD effect, comparing the GLS estimates to functional specific MR estimates

Type of variant selection	*nSNPs*	*Fixed effect*	*Random effects*
GLS	11	1.43 (0.89;2.29)	1.43 (0.89;2.29)
3 prime UTR variant	NA	NA	NA
intron variant	6	1.67 (0.80;3.50)	1.67 (0.80;3.50)
non coding transcript exon variant	NA	NA	NA
downstream gene variant	1	1.43 (0.07;28.14)	NA
missense variant	NA	NA	NA
upstream gene variant	3	1.35 (0.82;2.22)	1.35 (0.63;2.86)
splice region variant	NA	NA	NA
5 prime UTR variant	NA	NA	NA
synonymous variant	1	1.38 (0.48;3.91)	NA
Miss & splice	NA	NA	NA
3- & 5-UTR & intron & nc exon	6	1.67 (0.80;3.50)	1.67 (0.80;3.50)
Down & upstream	4	1.35 (0.82;2.22)	1.35 (0.72;2.52)

Supplementary Table 9: The *CETP* lipids - CHD effect, comparing the GLS estimates to functional specific MR estimates

Type of variant selection	*nSNPs*	*Fixed effect*	*Random effects*
GLS	26	0.84 (0.80;0.90)	0.84 (0.78;0.91)
3 prime UTR variant	1	1.04 (0.67;1.61)	NA
intron variant	17	0.83 (0.77;0.88)	0.83 (0.75;0.90)
non coding transcript exon variant	NA	NA	NA
downstream gene variant	NA	NA	NA
missense variant	1	0.92 (0.79;1.08)	NA
upstream gene variant	5	0.92 (0.84;1.00)	0.92 (0.84;1.00)
splice region variant	NA	NA	NA
5 prime UTR variant	1	2.56 (0.14;47.57)	NA
synonymous variant	1	1.24 (0.79;1.94)	NA
Miss & splice	1	0.92 (0.79;1.08)	NA
3- & 5-UTR & intron & nc exon	19	0.84 (0.79;0.89)	0.84 (0.76;0.92)
Down & upstream	5	0.92 (0.84;1.00)	0.92 (0.84;1.00)

Supplementary Table 10: Number of variants with eQTL and CHD data in a ± 2.5KB region around each locus; stratified by tissue (after clumping on $R^2 < 0.60$). GWAS data was extracted from GTEx [5].

	HMGCR	***PCSK9***	***NPC1L1***	***CETP***
Median (Q1;Q3) number of variants across tissues	1 (0; 28.25)	4 (1; 6)	2 (1; 19.5)	4.5 (2.75; 7.25)
Adipose Subcutaneous	33	1	1	8
Adipose Visceral Omentum	1	8	2	7
Adrenal Gland	21	2	18	4
Artery Aorta	1	70	2	3
Artery Coronary	20	1	1	4
Artery Tibial	0	3	45	53
Brain Amygdala	33	0	0	58
Brain Anterior cingulate cortex BA24	1	1	0	1
Brain Caudate basal ganglia	45	0	2	2
Brain Cerebellar Hemisphere	0	5	1	1
Brain Cerebellum	46	11	37	54
Brain Cortex	1	7	31	2
Brain Frontal Cortex BA9	61	2	2	2
Brain Hippocampus	1	0	0	1
Brain Hypothalamus	1	3	0	45
Brain Nucleus accumbens basal ganglia	1	4	0	53
Brain Putamen basal ganglia	1	0	1	40
Brain Spinal cord cervical c-1	32	3	1	3
Brain Substantia nigra	40	48	24	4

Breast Mammary Tissue	26	2	3	3
Cells EBV-transformed lymphocytes	48	0	47	9
Cells Transformed fibroblasts	0	5	2	9
Colon Sigmoid	45	4	1	1
Colon Transverse	1	4	0	7
Esophagus Gastroesophageal Junction	40	4	27	6
Esophagus Mucosa	1	1	1	3
Esophagus Muscularis	0	1	1	1
Heart Atrial Appendage	0	0	1	6
Heart Left Ventricle	0	0	1	1
Liver	36	4	41	7
Lung	1	6	1	7
Minor Salivary Gland	0	39	2	4
Muscle Skeletal	1	0	2	7
Nerve Tibial	0	6	2	3
Ovary	0	62	36	2
Pancreas	1	5	2	4
Pituitary	1	47	39	6
Prostate	0	1	1	9
Skin Not Sun Exposed Suprapubic	0	5	1	1
Skin Sun Exposed Lower leg	1	6	46	3
Small Intestine Terminal Ileum	0	4	53	8
Spleen	27	5	0	3
Stomach	1	3	47	7
Testis	1	12	2	5
Thyroid	1	0	1	5

Uterus	38	4	1	2
Vagina	1	4	2	64
Whole Blood	0	11	0	6

3 Supplementary Methods

Proteins as the exposure of interest

As addressed in manuscript under the mathematical derivations, the potential for horizontal pleiotropy (arising due to pathways proximal to the exposure of interest opening direct pathways to disease) increases the more distal the exposure of interest is from the genetic variants that are used as instruments. For example, Appendix Figure 7 depicts an MR study diagrammatically, where mRNA expression $\mathbf{E}$ causes a change in protein level $\mathbf{P}$, which in turn affects a downstream biomarker $\mathbf{X}$ and ultimately disease $\mathbf{D}$. We say horizontal pleiotropy is present when any node in the $\mathbf{G} \rightarrow \mathbf{D}$ path has a direct arrow to disease: ϕ. Below we discuss when such horizontal pleiotropy induces bias.

Here an MR of expression level effect on disease is unbiased by horizontal pleiotropy when $\phi_{\mathbf{G}} = 0$; requiring a relatively *weak* no horizontal pleiotropy assumption. Similarly, the causal effect of $\mathbf{P} \rightarrow \mathbf{D}$ can be determined under a slightly *stronger* assumption of no horizontal pleiotropy: $\phi_{\mathbf{G}} = 0$, and $\phi_{\mathbf{E}} = 0$. Finally, the causal effect of $\mathbf{X}$ on disease requires the *strongest* no horizontal pleiotropy assumptions $\phi_{\mathbf{G}}, \phi_{\mathbf{E}}, \phi_{\mathbf{P}} = 0$; that all direct pathways from the genetic variant, expression and protein are exactly zero. As such the more distal the inferential target from the genes, the more possibilities exist for horizontal pleiotropy.

As more thoroughly discussed in the main text, drug target MR is *often* concerned with assessing the potential causal effect(s) of a druggable protein. To delineate this we introduce the concepts of prior- and post-translational pleiotropy, where the former induces bias through horizontal pleiotropy, and the latter simply reflects a proteins ability to affect disease (both positive and negative) through multiple pathways. Comparing drug target MR to traditional MRs, often focussing on the role of downstream biomarkers such as $\mathbf{X}$, we can conclude that protein drug target MR has less potential for horizontal pleiotropy; The former needs $\phi_{\mathbf{G}}, \phi_{\mathbf{E}} \neq 0$, while latter needs to *additionally* assume $\phi_{\mathbf{P}} = 0$. Indeed the potential for bias inducing horizontal pleiotropy is greater still if the exposure lies further downstream a pathway of J many biomarkers; $\mathbf{X} = \{X_1, X_2, \cdots, X_J\}$.

Cis-instruments

To increase the plausibility of the absence of prior-translational pleiotropy drug target MR focus on *cis* genetic regions known to encode the protein drug target. Appendix Figure 8.a illustrates a *cis*-MR analysis where the causal effect of the protein of interest P_1 for a disease outcome is instrumented using SNPs in its encoding gene G_1. Proteins P_2 and P_3 are also altered by P_1 and hence are also associated with G_1 (an example of vertical pleiotropy), with P_2 being a mediator of the effect of P_1 on the disease outcome

and P_3 being a bystander. Instruments in G_1 are *cis-* for P_1 but *trans-* for P_2 and P_3, but this is immaterial in this case because the instrumented protein is P_1. The example illustrates how valid instruments for *cis*-MR can also have, and indeed would often times be expected to also have, *trans-* effects. However, this does not compromise the validity of *cis* instruments because the protein instrumented P_1 must be upstream of all of the other proteins in the causal pathway.

Panel b (Appendix Figure 2) illustrates a situation where P_1 is again the protein of interest and it remains causal for a disease outcome. However, in this analysis, the effect is instrumented by SNPs in a different gene G_1 encoding a protein P_4 which is unmeasured (i.e. a *trans*-MR analysis). Here P_4 affects P_1 via a receptor and signalling cascade. In this example, P_4 has no independent effect on disease outcome: its effect is through the protein of interest, P_1 For this reason, SNPs in G_4 also associate with P_2 and P_3 (vertical pleiotropy). In this example, a *trans*-MR analysis provides the correct inference on the causal relevance of P_1 for the disease outcome.

However, panel c illustrates the alternative situation where P_1 remains the protein of interest but this time it is not causal for the disease outcome. Its effect is again instrumented by SNPs in a different gene G_4 encoding an unmeasured protein P_4 (i.e. the same *trans*-MR analysis). However, in this scenario, P_4 affects disease outcome through a pathway independent of P_1. This time the association of SNPs in G_4 with P_1 (and P_2 and P_3) is due to horizontal pleiotropy and any inference that there is a causal association of P_1 with the disease outcome is erroneous. Notably, the situation in panel b (where P_1 is causal) and panel c (where it is not) are indistinguishable using *trans*-MR analysis: in both cases G_4 associates with P_1 (the protein of interest), P_2, P_3 and the disease outcome.

4 Supplementary Discussion

eQTL weighted drug target MR

Because tissue-specific eQTL data is more widely available than tissue-specific pQTL data, we additionally evaluated the performance of MR analysis using mRNA expression level as the exposure variable. An analysis of this type assumes a certain proportionality between mRNA and protein expression, but the strength of this assumption has not been tested systematically across human tissues. We note that [11] compared eQTLs and pQTLs in liver tissue from 192 outbred mice and found that 1400 of 6707 proteins studied had overlapping eQTLs and pQTLs, and among these overlapping proteins showed a high. Among proteins without shared eQTLs and pQTLs, the mean correlation was around 0.25. Recognising this caveat, we obtained information on genetic effects on mRNA expression from GTEx version 7, for (1MB) cis-regions of all four genes studied here, based on post mortem tissues from 449 donors (84% of European descent). Relative expression levels for each gene differed considerably across tissues and between each locus (Supplementary Figure 10). Most strikingly, *HMGCR* was

uniformly expressed across tissues, while *CETP* was most expressed in spleen, and PCKS9 and NPC1L1 in the liver. In Supplementary Figures 11-14 we provide tissue-specific eQTL estimates on a number of genetic regions, showing that *HMGCR* eQTL variants were located throughout the surrounding ±1MB region, and that associations for the other loci were more confined: *CETP* (±10KB), *PCSK9*(±250KB), and NPC1L1 (±250KB). There was also considerable directional inconsistency in the effects of *cis*-variants on expression across the various tissues, for example *NPC1L1* variants were negatively associated with expression in esophageal mucosa, with the same variants positively associated in aortic tissue (Supplementary Figure 13).

This directional inconsistency resulted in directionally discordant tissue-specific MR estimates of the same drug-target. For example, PCSK9 mRNA expression in the adrenal gland was associated with an increase in CHD risk: OR 1.09 (95%CI 1.02; 1.16), while PCSK9 expression in the uterus was associated with decreased CHD risk: OR 0.92 (95%CI 0.88; 0.97). Selecting variants from a broader ±1MB region universally attenuated effect estimates, with Egger correction (Supplementary Figure 15) moderately reversing this attenuation. Horizontal pleiotropy did not however fully explain the directional inconsistency in CHD effects across tissues. For example, HMGCR expression in the brain tissues (caudate basal ganglia, putamen basal ganglia, and spinal cord cervical c-1 tissues) suggest that increasing HMGCR prevents CHD (opposite the drug compound effect).

Region size

To explore the possible cause of this directional inconsistency we next explored the influence of genetic region by iteratively increasing the flanking region from ±2.5KB to ±1MB, selecting variants from upstream, downstream or in both direction of the gene. With all 4 loci showing similar behaviour, we focus here on the *CETP* region (see Supplementary Figures 16-19 for the other drug targets) finding that effect direction in any given tissue remained constant across the expanding region. The significance of the association with mRNA expression could either be attenuated when selecting variants from larger regions (e.g. lung tissue), or conversely increase, (e.g., spleen) potentially reflecting different regulatory regions including enhancers for the same gene in different tissues.

Statistical heterogeneity

To further explore the inconsistency in effect direction when weighting MR analysis by mRNA expression in different tissues, we estimated any potential causal relationship between mRNA expression level as the exposure and circulating lipid concentration (rather than CHD risk) as the outcome.

In these analyses, directional inconsistency was also observed (Supplementary Figure 20). However, comparing the mRNA expression level effect estimates on lipids, to the expression level estimates on CHD across tissues did not indicate a significant correlation between the two (Supplementary Figure 21; correlation estimates between -0.20 and 0.20). As expected both the CHD and lipids estimates showed a large degree of heterogeneity (Supplementary Figure 22), indicating either 1) remaining horizontal pleiotropy (despite Egger correction), or 2) true tissue-specific heterogeneity of the expression level effect on CHD. Excluding tissues or variants displaying heterogeneity, did not markedly decrease directional inconsistency in the estimated causal effects on lipids nor on CHD (Supplementary Figures 23-25).

Potential role of enhancer regions

Finally, to determine the influence of enhancer variants, we associated the number of tissue specific enhancers (extracted from [**Liu2017**]) to the MR results from the 4 positive control loci, which did not reveal any significant association (Supplementary Figure 26). Additionally, we explored if the number of enhancers could be associated to the direction of effect (encoded as a binary indicator) which did not show a significant association either (Supplementary Figure 27). In Supplementary Figure 28, we associated the tissue-specific heterogeneity statistics of the MR estimates to the distance to the nearest enhancer variants which again did not demonstrate a clear relationship.

5 Supplementary References

[1] The 1000 Genomes Project Consortium. "A global reference for human genetic variation." In: *Nature* 526 (7571 Oct. 2015), pp. 68–74. ISSN: 1476-4687. DOI: 10.1038/nature15393.

[2] Stephen Burgess, Frank Dudbridge, and Simon G Thompson. "Combining information on multiple instrumental variables in Mendelian randomization: comparison of allele score and summarized data methods." In: *Statistics in medicine* 35 (11 May 2016), pp. 1880–1906. ISSN: 1097-0258. DOI: 10.1002/sim.6835.

[3] Stephen Burgess, Verena Zuber, Elsa Valdes-Marquez, Benjamin B Sun, and Jemma C Hopewell. "Mendelian randomization with fine-mapped genetic data: Choosing from large numbers of correlated instrumental variables." In: *Genetic epidemiology* 41 (8 Dec. 2017), pp. 714–725. ISSN: 1098-2272. DOI: 10.1002/gepi.22077.

[4] Benjamin B Sun et al. "Genomic atlas of the human plasma proteome." In: *Nature* 558 (7708 June 2018), pp. 73–79. ISSN: 1476-4687. DOI: 10.1038/s41586-018-0175-2.

[5] GTEx Consortium. "Genetic effects on gene expression across human tissues." In: *Nature* 550 (7675 Oct. 2017), pp. 204–213. ISSN: 1476-4687. DOI: 10.1038/nature24277.

[6] Cristen J Willer et al. "Discovery and refinement of loci associated with lipid levels." In: *Nature genetics* 45 (11 Nov. 2013), pp. 1274–1283. ISSN: 1546-1718. DOI: 10.1038/ng.2797.

[7] Majid Nikpay et al. "A comprehensive 1,000 Genomes-based genome-wide association meta-analysis of coronary artery disease." In: *Nature genetics* 47 (10 Oct. 2015), pp. 1121–1130. ISSN: 1546-1718. DOI: 10.1038/ng.3396.

[8] Heribert Schunkert et al. "Large-scale association analysis identifies 13 new susceptibility loci for coronary artery disease." In: *Nature genetics* 43 (4 Mar. 2011), pp. 333–338. ISSN: 1546-1718. DOI: 10.1038/ng.784.

[9] Karsten Suhre et al. "Connecting genetic risk to disease end points through the human blood plasma proteome." In: *Nature communications* 8 (Feb. 2017), p. 14357. ISSN: 2041-1723. DOI: 10.1038/ncomms14357.

[10] Lisanne L Blauw et al. "CETP (Cholesteryl Ester Transfer Protein) Concentration: A Genome-Wide Association Study Followed by Mendelian Randomization on Coronary Artery Disease." In: *Circulation. Genomic and precision medicine* 11 (5 May 2018), e002034. ISSN: 2574-8300. DOI: 10.1161/CIRCGEN.117.002034.

[11] Joel M Chick et al. "Defining the consequences of genetic variation on a proteome-wide scale". In: *Nature* 534.7608 (2016), pp. 500–505.
