# Supplementary material for: Genetic drug target validation using Mendelian randomisation
Source: Nat Commun. 2020 Jun 26;11:3255. doi: 10.1038/s41467-020-16969-0 (PMC7320010; doi:10.1038/s41467-020-16969-0)
Supplement: Supplementary file 3 — Reporting Summary [file 41467_2020_16969_MOESM3_ESM.pdf]

nature research

Corresponding author(s): A F Schmidt

Last updated by author(s): 12-05-2020

Reporting Summary

Nature Research wishes to improve the reproducibility of the work that we publish. This form provides structure for consistency and transparency in reporting. For further information on Nature Research policies, see our Editorial Policies and the Editorial Policy Checklist.

Statistics

For all statistical analyses, confirm that the following items are present in the figure legend, table legend, main text, or Methods section.

n/a	Confirmed	
☐	☒	The exact sample size (*n*) for each experimental group/condition, given as a discrete number and unit of measurement
☐	☒	A statement on whether measurements were taken from distinct samples or whether the same sample was measured repeatedly
☐	☒	The statistical test(s) used AND whether they are one- or two-sided<br>*Only common tests should be described solely by name; describe more complex techniques in the Methods section.*
☒	☐	A description of all covariates tested
☐	☒	A description of any assumptions or corrections, such as tests of normality and adjustment for multiple comparisons
☐	☒	A full description of the statistical parameters including central tendency (e.g. means) or other basic estimates (e.g. regression coefficient) AND variation (e.g. standard deviation) or associated estimates of uncertainty (e.g. confidence intervals)
☐	☒	For null hypothesis testing, the test statistic (e.g. *F*, *t*, *r*) with confidence intervals, effect sizes, degrees of freedom and *P* value noted<br>*Give P values as exact values whenever suitable.*
☒	☐	For Bayesian analysis, information on the choice of priors and Markov chain Monte Carlo settings
☒	☐	For hierarchical and complex designs, identification of the appropriate level for tests and full reporting of outcomes
☐	☒	Estimates of effect sizes (e.g. Cohen's *d*, Pearson's *r*), indicating how they were calculated

Our web collection on statistics for biologists contains articles on many of the points above.

Software and code

Policy information about availability of computer code

Data collection	Data were collected from publicly available GWAS repositories; no specialist software was used for this.
Data analysis	All analyses were conducted using the R programming language (version 3.6.3 for Linux), with packages dplyr (0.8.5), ggplot2 (3.2.1), gridExtra (2.3), openxlsx (4.1.5), and wesanderson (0.3.6). Diagrams were programmed in TikZ (3.1.5b), and the appendix written in LaTeX (TeX Live version 2019) and knitr (1.28).

For manuscripts utilizing custom algorithms or software that are central to the research but not yet described in published literature, software must be made available to editors and reviewers. We strongly encourage code deposition in a community repository (e.g. GitHub). See the Nature Research guidelines for submitting code & software for further information.

Data

Policy information about availability of data

All manuscripts must include a data availability statement. This statement should provide the following information, where applicable:

- Accession codes, unique identifiers, or web links for publicly available datasets
- A list of figures that have associated raw data
- A description of any restrictions on data availability

All data are publicly available, see URL provided in the table below

Field-specific reporting

Please select the one below that is the best fit for your research. If you are not sure, read the appropriate sections before making your selection.

- [x] Life sciences
- [ ] Behavioural & social sciences
- [ ] Ecological, evolutionary & environmental sciences

For a reference copy of the document with all sections, see nature.com/documents/nr-reporting-summary-flat.pdf

Life sciences study design

All studies must disclose on these points even when the disclosure is negative.

Sample size	We did not perform any sample size calculations. Variants were selected based on LD-clumping .
Data exclusions	To improve coomputational stability, low MAF (below 0.01) variants were excluded (apriori defined).
Replication	Results were successfully replicated using 3 distinct exposure types.
Randomization	While genes cannot be randomly allocated, they are inherited in a random fashion (following Mendel's laws of inheritance).
Blinding	While subjects cannot be actively blinded from their genotype, most subjects would have been unaware of their genotype. Should they have been aware, the implication (if any) on disease would in most cases be insufficiently clear to result in any marked bias.

Reporting for specific materials, systems and methods

We require information from authors about some types of materials, experimental systems and methods used in many studies. Here, indicate whether each material, system or method listed is relevant to your study. If you are not sure if a list item applies to your research, read the appropriate section before selecting a response.

Materials & experimental systems

n/a	Involved in the study
x	Antibodies
x	Eukaryotic cell lines
x	Palaeontology and archaeology
x	Animals and other organisms
x	Human research participants
x	Clinical data
x	Dual use research of concern

Methods

n/a	Involved in the study
x	ChIP-seq
x	Flow cytometry
x	MRI-based neuroimaging
